# Marker-free quantification of repair pathway utilization at Cas9-induced double-strand breaks

**DOI:** 10.1093/nar/gkab299

**Published:** 2021-05-08

**Authors:** Wanjuan Feng, Dennis A Simpson, Jang-Eun Cho, Juan Carvajal-Garcia, Chelsea M Smith, Kathryn M Headley, Nate Hathaway, Dale A Ramsden, Gaorav P Gupta

**Affiliations:** Lineberger Comprehensive Cancer Center, University of North Carolina, Chapel Hill, NC 27599, USA; Lineberger Comprehensive Cancer Center, University of North Carolina, Chapel Hill, NC 27599, USA; Lineberger Comprehensive Cancer Center, University of North Carolina, Chapel Hill, NC 27599, USA; Lineberger Comprehensive Cancer Center, University of North Carolina, Chapel Hill, NC 27599, USA; Curriculum in Genetics and Molecular Biology, University of North Carolina, Chapel Hill, NC 27599, USA; Biological and Biomedical Sciences Program, University of North Carolina, Chapel Hill, NC 27599, USA; Lineberger Comprehensive Cancer Center, University of North Carolina, Chapel Hill, NC 27599, USA; Biological and Biomedical Sciences Program, University of North Carolina, Chapel Hill, NC 27599, USA; School of Pharmacy, University of North Carolina, Chapel Hill, NC 27599, USA; School of Pharmacy, University of North Carolina, Chapel Hill, NC 27599, USA; Lineberger Comprehensive Cancer Center, University of North Carolina, Chapel Hill, NC 27599, USA; Department of Biochemistry and Biophysics, University of North Carolina, Chapel Hill, NC 27599, USA; Lineberger Comprehensive Cancer Center, University of North Carolina, Chapel Hill, NC 27599, USA; Department of Biochemistry and Biophysics, University of North Carolina, Chapel Hill, NC 27599, USA; Department of Radiation Oncology, University of North Carolina, Chapel Hill, NC 27599, USA

## Abstract

Genome integrity and genome engineering require efficient repair of DNA double-strand breaks (DSBs) by non-homologous end joining (NHEJ), homologous recombination (HR), or alternative end-joining pathways. Here we describe two complementary methods for marker-free quantification of DSB repair pathway utilization at Cas9-targeted chromosomal DSBs in mammalian cells. The first assay features the analysis of amplicon next-generation sequencing data using ScarMapper, an iterative break-associated alignment algorithm to classify individual repair products based on deletion size, microhomology usage, and insertions. The second assay uses repair pathway-specific droplet digital PCR assays (‘PathSig-dPCR’) for absolute quantification of signature DSB repair outcomes. We show that ScarMapper and PathSig-dPCR enable comprehensive assessment of repair pathway utilization in different cell models, after a variety of experimental perturbations. We use these assays to measure the differential impact of DNA end resection on NHEJ, HR and polymerase theta-mediated end joining (TMEJ) repair. These approaches are adaptable to any cellular model system and genomic locus where Cas9-mediated targeting is feasible. Thus, ScarMapper and PathSig-dPCR allow for systematic fate mapping of a targeted DSB with facile and accurate quantification of DSB repair pathway choice at endogenous chromosomal loci.

## INTRODUCTION

DNA double strand breaks (DSBs) represent a major threat to genome integrity (1). Unrepaired DSBs can result in cell death, whereas imprecise DSB repair promotes genome instability and tumorigenesis. DSB repair also determines the outcomes of clastogenic cancer therapies, as well as Cas9-mediated genome engineering (2). Thus, the regulation of DSB repair contributes to health, disease, and DNA-directed therapies.

Cells use two canonical pathways to repair chromosomal DSBs: Homologous Recombination (HR) and Non-Homologous End Joining (NHEJ) (3). A third DSB repair pathway is often referred to as alternative end joining (alt-EJ) or microhomology-mediated end joining (MMEJ). It is typically characterized by its independence from NHEJ, a requirement for resected breaks and resulting 3′ ssDNA overhangs, and favors the generation of deletions at short tracts (i.e. 2–6 bp) of sequence identity (microhomologies) that flank the DSB (4). The majority of cellular alt-EJ/MMEJ repair in metazoans and plants can be attributed to the activity of DNA Polymerase Theta (Pol θ, gene name *POLQ*), thus we and others define this pathway as Theta mediated end joining (TMEJ) (5,6). How and when TMEJ is utilized for chromosomal DSB repair remains enigmatic due to a lack of assays that assess pathway activity with high specificity.

The analysis of chromosomal DSB repair products after inducible expression of a site-specific endonuclease, or more recently Cas9, has been instrumental in the characterization of DSB repair pathway utilization in mammalian cells (7,8). Engineered DNA constructs are cleaved by a nuclease to generate a two-ended DSB, and specific repair outcomes—typically corresponding to a DSB repair pathway of interest (e.g. HR or NHEJ)—result in activation or loss of reporter gene expression (e.g. fluorescent protein or drug resistance gene). Reporter constructs for different DSB repair pathways of interest have been designed and validated, including homologous recombination (HR), single strand annealing (SSA), NHEJ, and MMEJ (9–14). Despite their undisputed utility, reporter-based DSB repair assays also have some limitations. This includes the requirement to generate a stably integrated reporter, thus limiting feasibility of use in primary cells (15), and the lag between generation of repair products and expression of the marker that confounds analysis of repair kinetics.

High-throughput amplicon next generation sequencing (NGS) across Cas9-induced DSBs can reveal the spectrum of repair products generated at a locus of interest. Several bioinformatics pipelines have been developed to quantify mutagenized repair outcomes (16–19). The majority of these algorithms use a score-based alignment of the entire amplicon to the reference sequence to identify insertions, deletions, and single nucleotide variants, followed by secondary interpretation of this alignment to generate statistics on the abundance of individual repair outcomes. Such analyses have been useful for evaluating the efficiency of gene conversion and for predicting dominant repair outcomes to facilitate genome engineering (19–22). However, score-based alignment algorithms can perform unpredictably as deletion or insertion size increases, and particularly when there is a combination of deletions and insertions at the junction site. This limitation of currently available bioinformatic pipelines results in incomplete characterization of repair product spectra when analyzing amplicon sequencing data. Although most of the available pipelines efficiently quantify the frequency of gene conversion at the target locus, the further classification of the non-HR repair products for end-joining repair patterns requires manual interpretation. Thus, comprehensive evaluation of HR, NHEJ and MMEJ/TMEJ repair patterns at Cas9-targeted loci using NGS remains a challenge.

In this study, we present two complementary methods to quantitatively profile HR, NHEJ, and MMEJ/TMEJ repair outcomes at endogenous Cas9-induced chromosomal breaks in mammalian cells. First, we describe a custom Python script, ScarMapper, that enables automated analysis of DSB repair patterns at Cas9-targeted chromosomal loci using NGS of target site-containing amplicons. Next, we illustrate how ScarMapper amplicon NGS analyses can be used to design pathway product-specific dPCR assays, PathSig-dPCR, for absolute quantification of signature HR, NHEJ, and TMEJ repair outcomes in unlabeled mammalian cells. We apply these assays to measure the impact of various genetic and pharmacologic perturbations on DSB repair pathway choice at a chromosomal DSB. We propose that the combination of ScarMapper analysis of amplicon sequencing and PathSig-dPCR can be applied to nearly any chromosomal locus of interest to better understand context-specific regulation of DSB repair pathway utilization in mammalian cells.

## MATERIALS AND METHODS

### Cell lines

Isogenic SV-40 large T antigen immortalized mouse embryo fibroblasts (MEFs) that are wild type (*WT)*, *Ku70^−^^/^^−^*, *Brca2* deficient (*Brca2^m/m^*), *Polq^−^^/^^−^*, and a subclone of *Polq^−^^/-^*complemented by expression of human POLQ (*Polq^hPOLQ^*) were previously described (6,23). MEFs expressing DR-GFP reporter were a gift from Dr. Jeremy Stark lab (24). All MEFs were maintained in Dulbecco's modified Eagle's medium (DMEM, Corning), with 10% Bovine Calf Serum (Hyclone BCS) and 2 mM l-glutamine (ThermoFisher), and *Polq^hPOLQ^* cells were supplemented with 2 μg/ml puromycin. Mouse ES cells (TC1) were cultured with DMEM supplemented with 15% FBS (Gibco, 1972526), 10 mM HEPES pH 7.5, 10 mM NEAA, 0.1% 2-betamercaptoethanol (Gibco, 21985023), 1% Penn-Strep (Corning, 30-002-CI) and 1:500 Leukemia inhibitory factor (LIF) treated media produced from LIF-1Cα (COS) cells. All cells were maintained at 37°C in an atmosphere of 5% CO_2_. Cells in culture were routinely monitored for mycoplasma contamination using the Plasmo Test™ (Invivogen).

### Transfection

For MEFs, 2.5 million cells were transfected with 5 μg Flag-Cas9 (A gift of Xingxu Huang, Addgene # 44758), 5 μg sgRosa26 (6), with or without 20 μg HR donor (HRD-500 see ref(6); HRD-200, see Supplementary Figure S4C) and 1 μg pEGFP-N2 (Takara) with a Neon transfection kit (Invitrogen) using a 1350 V, 30 ms pulse in a 100 μl chamber. Forty-eight hours post transfection, a portion of the cells were analyzed by flow cytometry to quantify the transfection efficiency, and the remaining cells were collected for genomic DNA extraction (Qiagen, DNeasy Blood & Tissue kits). For high throughput sequencing and time course experiments, we performed a Neon transfection using a Cas9 ribonucleoprotein (Cas9-RNP) assembled from Cas9 protein and RNA that targets Cas9 nuclease activity to a site in the Rosa26 locus (Alt-R system; IDT Integrated DNA Technologies).

For mouse ES cells (TC1), 0.325 ug flag-Cas9, 0.325 ug sgRosa26, 1.3 ug HRD-500 and 0.065 ug N2-EGFP were co-transfected into 2 million cells using Amaxa 4D Nucleofector (P3 Kit) on program CY104. Cells were treated with DNA-PKi NU7441 (5μM), or DMSO for 65 h. DNA was extracted for digital PCR on Day 7.

For MEFs DR-GFP reporter assay, we performed a Neon transfection using Cas9-RNP-sgRosa26 and Cas9-RNP-sgDRGFP (Alt-R system; IDT Integrated DNA Technologies).

For MEFs AR-TLR, 2.5 million cells were transfected with 5 μg I-Sce-BFP (a gift from Andrew Scharenberg, Addgene #45574) and 5 μg d14GFP (a gift from Andrew Scharenberg, Addgene #31475) with a Neon transfection kit (Invitrogen) using a 1350 V, 30 ms pulse in a 100 μl chamber. Forty-eight hours post transfection, cells were analyzed by flow cytometry according to previous description (25).

### Generation of MEFs expressing the AR-TLR reporter

Lentivirus were generated by 293T cells in 150mm dish with transfection of 3 μg pMD2.G (a gift from Didier Trono, Addgene #12259), 4.5 μg psPAX2 (a gift from Didier Trono, Addgene #12260) and 6 μg AR-TLR (a gift from Andrew Scharenberg, Addgene #45575). Transfection was performed using polyethylenimine (Polysciences, Inc). Supernatant was collected at 48h and filtered with 0.45 μM filter. Forty-eight hours after transduction, MEFs were sorted for iRFP713 by FACSAria2.

### High throughput sequencing

Genomic DNA was harvested 24 h after introduction of Cas9 targeted to the Rosa26 locus for amplicon-based library preparation. Amplification primers contained 3′ locus specific sequence as well as indexes to allow for library multiplexing. Base-calling in Illumina platforms is less effective when many reads have identical sequence (low sample complexity), thus we employed primer variants with different numbers of inserted nucleotides (phasing nucleotides) between locus specific sequence and the Illumina sequencing primers (26,27). The resulting multiplexed paired-end FASTQ files were analyzed using ScarMapper.

To prepare the libraries, 200 ng of genomic DNA from each sample was divided between two PCRs containing 250 nM of the forward and reverse primer or primer pool, 200 μM dNTPs, Phusion™ polymerase HF buffer and 1 μl of Phusion™ polymerase (New England BioLabs). The PCR cycling conditions are shown in Supplemental Methods. The primary PCR product of the Rosa26 locus is 482–504 bp while that of the lamin receptor B (*LBR*) locus is 275–285 bp. The products were purified from the unincorporated primers and genomic DNA using a two-step SPRI bead (Ampure, Beckman Coulter or sparQ pureMag, VWR) extraction. Once purified, 20–30 ng of each of these products were used to add the Illumina P5 and P7 adapters (*Rosa26* set) or Illumina dual indexing adapters (*LBR* set) in a 50 μl PCR reaction according to the conditions in Supplemental PCR Conditions. These products were purified using a single step SPRI bead extraction with a 1:1 ratio of PCR product to beads. Following purification to remove unincorporated primers and primer dimers the products were quantified using a Qubit™ and then pooled for sequencing. Pooled libraries were subjected to 2 × 300 cycles on an Illumina MiSeq (*Rosa26* locus) or 2 × 150 cycles on an Illumina iSeq100 (*LBR* locus).

### Repair scar mapping

ScarMapper (https://github.com/Gaorav-Gupta-Lab/ScarMapper.git), the custom Python program developed for this method, is run according to the user guide (ScarMapper User Guide.docx). When using overlapping paired end reads Read 1 and the reverse complement of read 2 are merged to generate a consensus read. Multiplexed, paired end FASTQ files from the sequencing were analyzed with ScarMapper to quantify the different patterns of break repair observed in each library sample. The different repair patterns observed were binned into a repair type using the following definitions. NHEJ is defined as products with deletion size <4 nucleotides and insertion size <5 nucleotides. Non-Microhomology Deletions are products containing deletion size ≥4 nucleotides, insertion size <5 nucleotides, and microhomology (MH) <2 nucleotides. Theta mediated End Joining (TMEJ) is defined as products with deletion size ≥4 nucleotides and MH ≥2 nucleotides. Insertions classification is defined as products with insertions ≥5 nucleotides. Homologous recombination (HR) products are identified by the presence of a user-defined gene conversion sequence (from the HR donor) at the junction site.

### Droplet digital PCR

Droplet digital PCR (dPCR) was performed according to dPCR™ Supermix for Probes (No dUTP) instruction. In general, 25 μl reactions were made to include 1× dPCR Supermix for Probes (No dUTP), 900 nM each primer, 250 nM probe (FAM or HEX) and 80ng of genomic DNA with dH_2_O into per reaction. An automated droplet generator (Bio-Rad) transferred 20 ul of these reactions to first generate emulsions, then aliquot emulsions to a new 96-well plate. Emulsified reactions were subject to PCR amplification using a C1000 thermal cycler (Bio-Rad), followed by analysis using the QX200 droplet reader. PCR thermocycling conditions for the different amplicon regions evaluated in this study are provided in Supplementary Methods. The repair signal was normalized to 100 copies of genomic DNA as measured using the Chromosome 6 control dPCR assay. All the primers and probes are listed in Primers and probes for dPCR in Supplementary Table S1. Analysis of dPCR data was performed using QuantaSoft (Bio-Rad).

### Antibodies and chemicals

Antibodies against Mre11 (NBP2-59677) and Rad50 (NBP2-20054) were purchased from Novus Biologicals. Antibody against Nbs1 was described previously (28). DNA-PK inhibitor (NU7441) was purchased from Selleck Chemicals.

## RESULTS

### ScarMapper methodology

Amplicon paired-end next-generation sequencing (NGS) is commonly used to evaluate repair outcomes at a Cas9-targeted chromosomal DSB. After generating a consensus read from the paired-end reads, a majority of the currently available bioinformatic pipelines proceed to identify and assign insertions and deletions (indels) after alignment of the consensus read to the uncut reference, using algorithms (e.g. Bowtie, Needleman-Wunsch) that are largely unoriented to the known Cas9 cleavage site (16–18,29). Such methods do not take advantage of the ability to infer that indels associated with repair—or at least end-joining repair—must originate at the site of the DSB. These methods consequently often inappropriately locate complex insertion and deletion events adjacent to the site of the DSB, especially when deleted sequence is significantly greater on one side of the break, relative to the other. Further limiting the utility of methods that align the entire read to an uncut reference is the overweighting of gap penalties. This results in misclassification of complex indels as single nucleotide variants, especially when junctions have large (>4 bp) deletions. Thus, the classification of NGS reads based on different DSB end-joining repair patterns often requires manual inspection and interpretation. ScarMapper was designed to overcome these limitations and thereby enable automated analysis of HR and end joining repair patterns at Cas9-targeted chromosomal DSBs by NGS.

**Figure 1.**
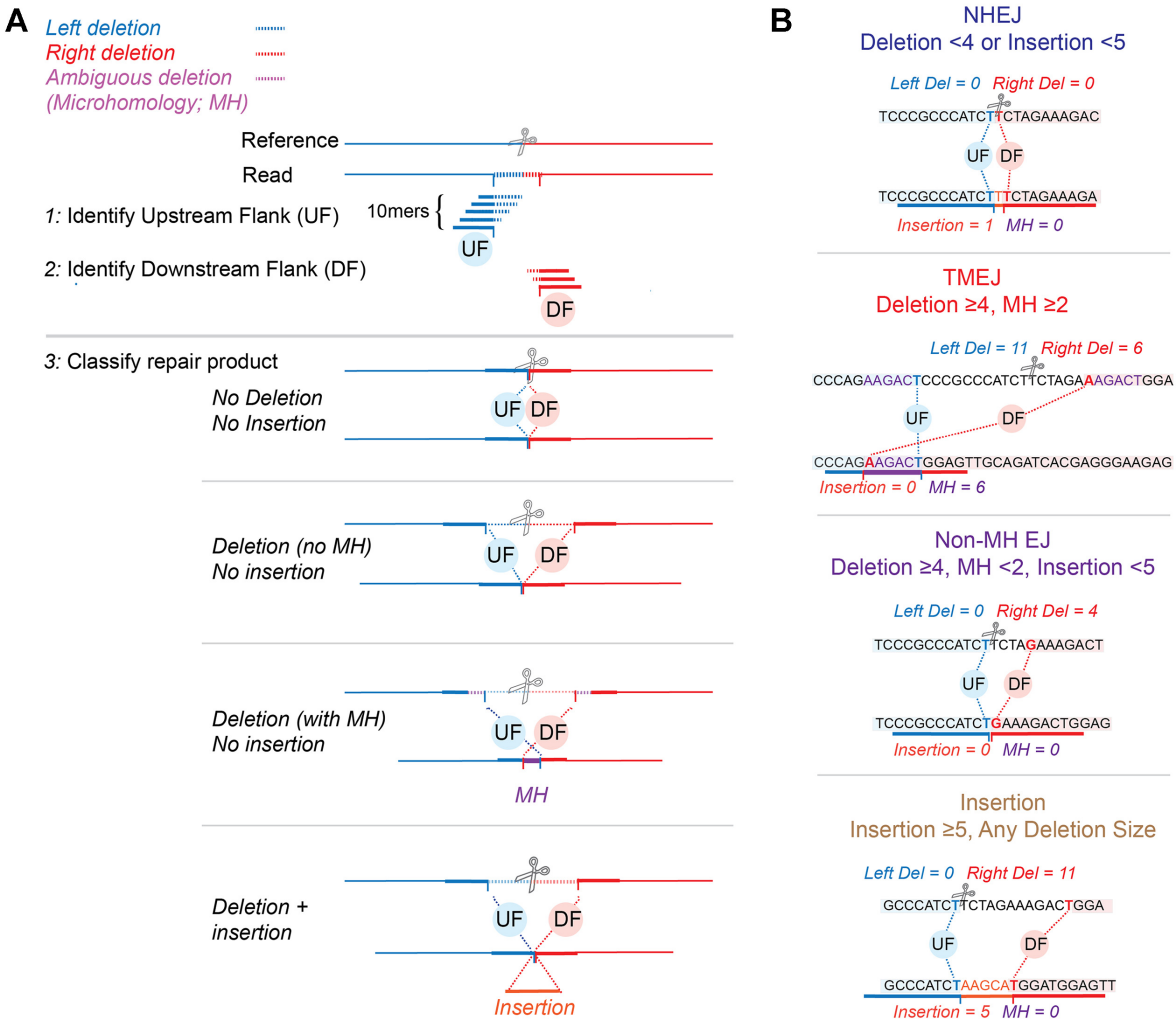
ScarMapper analysis pipeline. (**A**) The target region of a Cas9 generated DSB (scissors) is amplified and sequenced. Blue and red lines denote sequence upstream and downstream of the DSB site and is represented as a dashed line in the consensus read when deleted, relative to the reference. Orange lines represent inserted sequence. Junctions at sites where one copy of identical sequence is deleted (microhomology; MH) have the microhomology represented in purple. Step 1: ScarMapper generates a dictionary of 10 mers consistent with increasing deletion in 1 nt increments of upstream sequence flanking the cas9 target site. The upstream flank (UF) is then located in each consensus read by the identification of a matching 10mer from this dictionary that has the least amount of upstream flank deletion. In step 2, the same process is used to identify the downstream flank (DF). In step 3, each read is classified as having deletions, deletions with microhomology, or insertions (with or without deletion), then further assigned to repair pathways using pathway definitions as noted in (**B**).

ScarMapper (Figure 1) employs a k-mer based method, which iteratively tests for matches to a sliding window of 10-mers that represent candidate upstream and downstream flanks (UF, DF). It first identifies within each consensus read the 10-nucleotide match consistent with the least amount of deletion of sequence upstream of the break site, to determine the UF (Figure 1A, step 1). It then performs the same method to identify the 10 nucleotide match consistent with the least amount of deletion of sequence downstream of the break site, to determine the DF (Figure 1A, step 2). Junctions with perfectly abutted UF and DF 10-mers defined this way describe simple deletions, overlap between UF and DF 10-mers describe a microhomology associated with a deletion, and sequence between UF and DF are insertions (Figure 1A, step 3). The Left-Deletion and Right-Deletion sizes are readily measured as the distance from the UF and DF, respectively, from the known Cas9 cleavage site in the reference sequence (Figure 1A, step 3). The total deletion size is calculated as the sum of the Left-Deletion, Right-Deletion, and MH size. This method has additional advantages over conventional mapping methods as it is insensitive to sequencing errors when these errors are distal to the 20 nucleotides used to define the junction, and its fidelity in characterizing junctions is not affected by the size of insertions and deletions.

ScarMapper outputs a database of observed repair products based on four features: (i) left deletion size, (ii) right deletion size, (iii) microhomology sequence and (iv) insertion sequence. Normalized frequencies and absolute read counts are tabulated for each repair product. Non-mutated reads are excluded from the repair product frequency analyses, as a large fraction of these reads are attributable to cells where the chromosome was not cut by Cas9. If a homology donor was included in the experiment, the frequency of the HR repair product is also reported. ScarMapper classifies all of the non-HR repair products into four groups (Figure 1B). ‘NHEJ’ is defined as repair products with deletions <4 nucleotides and insertions < 5 nucleotides. Repair products with deletions ≥4 nucleotides and microhomology (MH) ≥2 nucleotides are classified as Theta Mediated End Joining (‘TMEJ’), based on prior demonstration of polymerase theta dependency (26). Non-MH end joining ‘Non-MH EJ’ is defined as deletions ≥4 nucleotides, MH <2 nucleotides, and insertion <4 nucleotides. ‘Insertion’ is defined as insertion ≥5 nucleotides, regardless of associated deletion size. These patterns encompass all the repair products observed.

### ScarMapper analysis of the murine *Rosa26* locus

**Figure 2.**
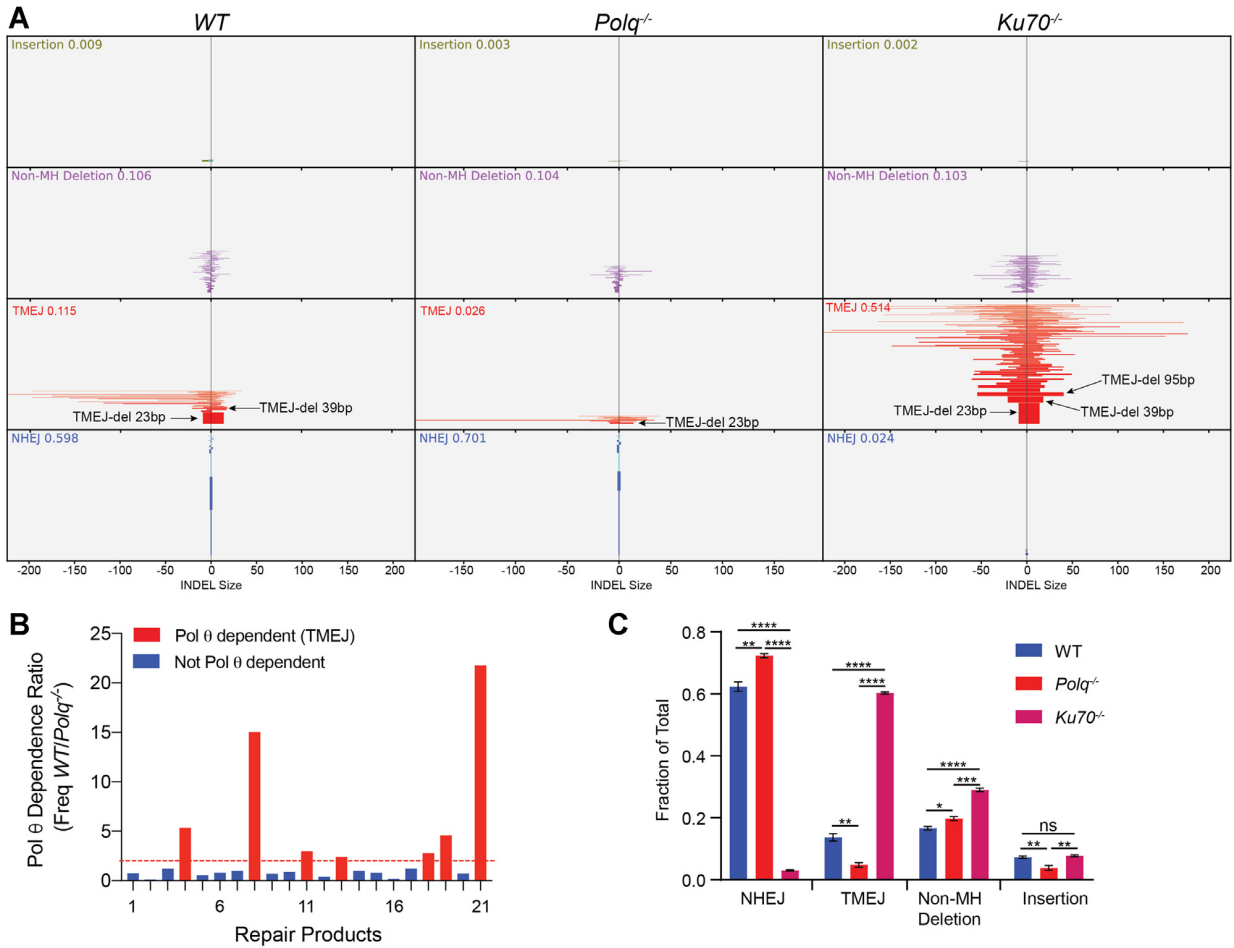
ScarMapper Analysis of the Murine Rosa26 Locus. (**A**) ScarMapper graphical output of Rosa26 locus paired-end NGS data for *WT* (left panel), *Polq*^–/–^ (middle panel), and *Ku70^–/–^* (right panel) MEFs. The x-axis is the number of nucleotides deleted and/or inserted to the left or right of the Cas9 cut site. The values in each scar type are ranked according to the allelic frequency. The height of the bars is proportional to the frequency. The plots show frequencies ≥0.00025. (**B**) Pol θ dependency of scar patterns. The allelic frequency ratio (*WT*/*Polq^−^^/^^−^*) for the 21 most prevalent repair products observed in *WT* cells. Red bars indicate repair products that are Pol θ-dependent (ratio ≥2). (**C**) Histogram of scar types in the different cell lines. Mean ± SEM are shown. Significance by two-tailed unpaired Student's *t*-test. *P <0.05; **P <0.02; ***P <0.002; ****P <0.0001.

ScarMapper was applied to a dataset of paired-end amplicon sequencing data obtained after transfection of *WT*, *Polq^−^^/^^−^* and *Ku70^−^^/^^−^* MEFs with Cas9-RNP targeting the *Rosa26* locus (6,23). An example of ScarMapper applied to the human Lamin B receptor (*LBR*) locus is shown in Supplementary Figure S1. Each broad group of repair products is clustered based on the aforementioned repair pathway classification for visual comparison across the cell types (Figure 2A). The primary bar represents the size of the deletion observed on the upstream (left of centerline) or downstream (right of centerline) flanks of the cut site. Within each group the patterns are ordered according to their frequency. The height of each bar in the histogram is proportional to the normalized frequency for that repair product within the population. As anticipated, *Polq^−^^/^^−^* MEFs have reduced TMEJ, whereas *Ku70^−^^/-^* MEFs exhibit both reduced NHEJ and a compensatory increase in TMEJ (Figure 2A). However, not all TMEJ repair products are increased equally; a 23 bp deletion product with 6 bp MH (TMEJ-23bp) doubled (6.2–12.4%) in *Ku70^−^^/^^−^* MEFs, while two larger deletions—both a 39 bp deletion with 4 bp MH (TMEJ-39 bp, 0.5–3.8%) and a 95bp deletion with 5 bp MH (TMEJ-95 bp, below limit of detection to 2.3%)—are substantially increased in *Ku70^−^^/^^−^* MEFs, relative to WT. This bias towards TMEJ products with larger deletion sizes is likely due to hyper-resection of DSB ends, which is a known phenotype associated with Ku deficiency (30). As anticipated, NHEJ deficiency in *Ku70^−^^/^^−^* MEFs was also associated with substantially higher rates of HR repair, which was readily quantified by ScarMapper (Supplementary Figure S2).

Evaluation of Pol θ dependency may be desired to define TMEJ repair products more precisely, as illustrated in a recent study of Pol θ-mediated genomic scars (26). If a *Polq*-deficient control is available, ScarMapper enables repair product-specific analysis of Pol θ-dependency (Freq in sample/Freq in *Polq*-deficient control) in the output file. Analysis of Pol θ dependence was performed on the *Rosa26* locus NGS data to identify repair products with a Theta-dependency ratio >2 (Figure 2B).

### Comparison of ScarMapper and CRISPRESSO2

The performance of ScarMapper was compared to CRISPResso2 using the same FASTQ files of the murine *Rosa26* locus as input (16). There was excellent concordance across both platforms when measuring allelic frequency for ‘simple’ deletions that lacked any associated insertion sequences (Supplementary Figure S3A). However, detection of repair products with insertions was highly discordant, with ScarMapper identifying 363 insertion patterns in the *Ku70^−^^/^^−^* cells, while CRISPResso2 only detected 85 (Supplementary Figure S3B). Examination of representative complex repair products (containing both insertions and deletions) from the *Ku70^−^^/^^−^* genotype illustrates the difficulty CRISPResso2 has in aligning these to the reference, whereas ScarMapper is able to clearly demarcate the position of upstream/downstream deletions and inserted sequences (Supplementary Figure S3C-D). In addition to these alignment-related limitations, CRISPResso2 does not provide any information regarding microhomology usage, nor does it classify repair products into functional categories to facilitate visualization and data interpretation. Thus, ScarMapper enables more comprehensive and automated interpretation of Cas9-induced DSB repair patterns by amplicon sequencing, relative to currently available analysis programs (16,18,19).

### Absolute quantification of specific repair products using PathSig-dPCR

**Figure 3.**
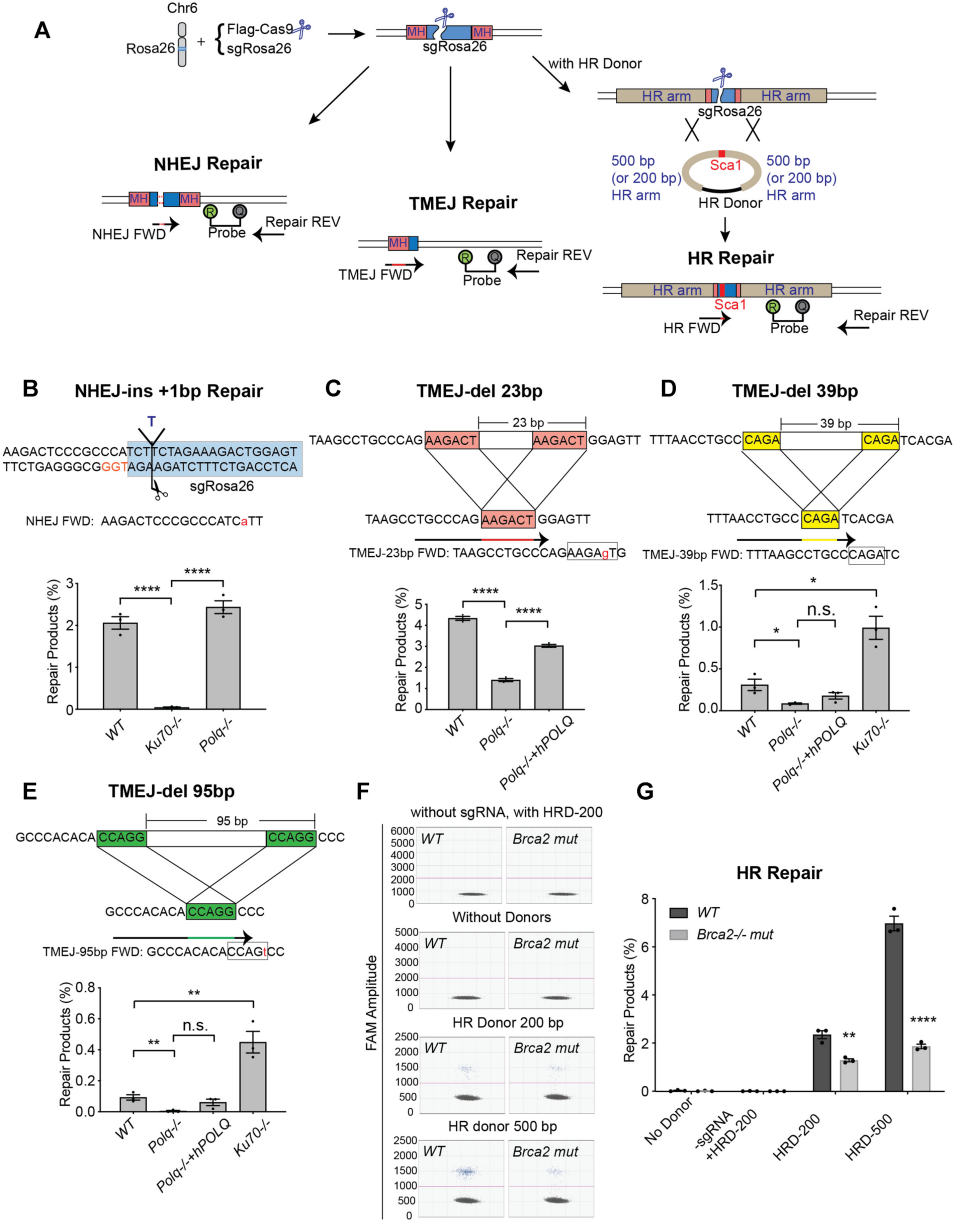
PathSig-dPCR assay designs to monitor NHEJ, TMEJ and HR repair at the murine Rosa26 locus. (**A**) Schematic diagram of PathSig-dPCR assays applied to the murine Rosa26 locus. Cells are transfected with Flag-Cas9 and sgRosa26 by Neon electroporation. 24–48 hours post transfection, genomic DNA is assayed by dPCR for signature products reflecting repair by NHEJ, TMEJ and HR. (**B**) NHEJ repair - one ‘T’ insertion repair product was validated in *WT*, *Ku70^−^^/^^−^* and *Polq^−^^/^^−^* cells by dPCR. The NHEJ forward primer includes a ‘T’ to ‘A’ mismatch to destabilize the primer against amplification of the wild-type sequence. (**C-E**) Three TMEJ repair products were validated by dPCR. (**C**) Signature of TMEJ-del 23 bp was validated in *WT*, *Polq^−^^/^^−^* and *Polq^−^^/^^−^* + *hPOLQ* cells. TMEJ-del 39 bp (**D**) and TMEJ-del 95 bp (**E**) were validated in *WT*, *Polq^−^^/^^−^, Polq^−^^/^^−^* + *hPOLQ* and *Ku70^−^^/^^−^* cells. (**F**) Scatter plots of droplets showing HR events induced by Rosa26 cut site. (**G**) HR repair products were validated by adding 200 bp HR donor (HRD-200) or 500 bp HR donor (HRD-500) in *WT* and *Brca2^−^^/^^−^* mutant cells. Mean ± SEM are shown. Statistical significance was assessed by two-tailed *t*-tests. *P <0.05, ** *P* <0.01 and **** *P* <0.0001.

To more robustly measure the abundance of specific DSB repair products, we used ScarMapper analysis of the *Rosa26* locus to first identify products that are characteristic of repair by different pathways. We then designed droplet digital PCR (dPCR) assays specific for these products (Figure 3A), including one for NHEJ (+1 insertion, Figure 3B and Supplementary Figure S4A), three for TMEJ (23, 39 and 95 bp deletions, Figures 3C–E and Supplementary Figure S4B), and one for HR events generated by gene targeting in the presence of a double stranded donor with either 200 bp (HRD-200) or 500 bp (HRD-500) homology arms (Figure 3A, F, G and Supplementary Figure S4C). Selectivity for these repair products was achieved by spanning the forward primer across the junction and in some cases additionally destabilizing the 3′ terminus through introduction of a near terminal mismatch (Figure 3B–D, Supplementary Figures S5 and S6). An additional dPCR amplicon was designed for a genomic locus 29 kb upstream of the Rosa26 locus on chromosome 6 and was used to normalize the observed abundance of a particular repair product relative to 100 copies of this reference amplicon; all data was then represented as ‘Percentage Repair’. Collectively, we refer to this approach as ‘PathSig-dPCR’ because it enables absolute quantification of DSB repair by NHEJ, TMEJ, and HR in cells after induction of a chromosomal DSB by Cas9.

PathSig-dPCR analysis was performed on genomic DNA harvested from MEF cell lines 48 hours after transfection with Cas9 and *Rosa26* locus-targeting sgRNA. PathSig-dPCR reveals exquisite dependence of the NHEJ repair product on *Ku70*, diminishing over 50-fold in *Ku70^−^^/^^−^* MEFs (0.04% allele frequency) relative to *WT* and *Polq^−^^/^^−^* MEFs (2% and 2.4% of detected loci, respectively) (Figure 3B and Supplementary Figure S4A). We next analyzed DNA from *WT*, *Polq^−^^/^^−^* and reconstituted *Polq^−^^/^^−^ + POLQ* (*Polq^POLQ^*) cells for TMEJ-del 23 bp, TMEJ-del 39 bp, and TMEJ-del 95 bp products (Figure 3C–E and Supplementary Figure S4B). The PathSig-dPCR signal for TMEJ-del 23 bp, and TMEJ-del 39 bp products were 4.3% and 0.3% in WT cells and reduced to 1.4% and 0.09% in *Polq^−^^/^^−^* cells. Reconstitution of *POLQ* expression in *Polq^−^^/^^−^* MEFs (*Polq^POLQ^*) restored TMEJ-del 23 bp, and TMEJ-del 39bp products back to 3% and 0.18%, respectively. The rare event TMEJ-del 95bp had a frequency of 0.09% in WT cells and 0.004% in *Polq^−^^/^^−^* cells. Deficiency in *Ku70* resulted in increases in frequency for all TMEJ products, but these increases were proportionally greater for TMEJ products with larger deletion (Figure 3D, E), consistent with an increase in TMEJ repair and a hyper-resection phenotype that is associated with larger-sized deletions. PathSig-dPCR also confirmed that HR rates are increased for HRD-500 (7%) relative to HRD-200 (2.4%), and that both are reduced in MEFs expressing a mutant *Brca2* allele (Figure 3F, G). Thus, PathSig-dPCR is a highly tractable and accurate method for precisely measuring repair by NHEJ, TMEJ, and HR after Cas9-directed chromosomal DSBs.

### Inhibition of End Resection or DNA-PK alters DSB repair pathway utilization

**Figure 4.**
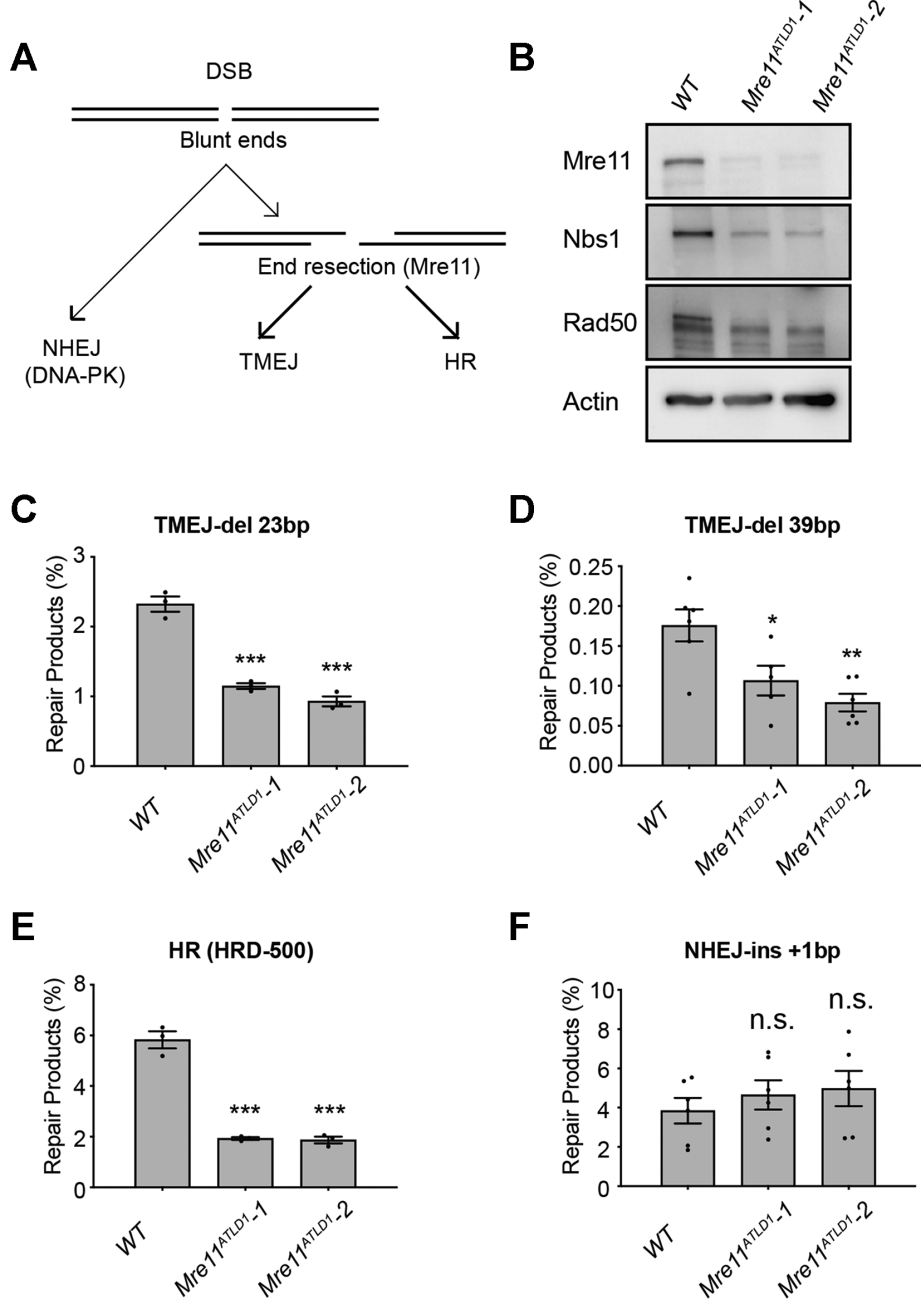
Mre11 hypomorphism alters DSB repair pathway utilization. (**A**) Schema depicting the role of Mre11-mediated end resection in the regulation of DSB repair pathway choice. (**B**) *Mre11^ATLD1/ATLD1^* MEFs exhibit reduced expression of the MRN complex by immunoblotting. (**C–F**) DSB repair products were identified in *WT*, *Mre11^ATLD1^*-1 and *Mre11^ATLD1^*-2 cells. (**C**) TMEJ-del 23 bp. (**D**) TMEJ-del 39 bp. (**E**) HR repair (HRD-500 donor) and (**F**) NHEJ-ins +1 bp. Mean ± SEM are shown. Statistical significance was assessed by two-tailed *t*-tests. * *P* < 0.05, ** *P* < 0.01, *** *P* < 0.001 and **** *P* < 0.0001.

DSBs can trigger nucleolytic resection of broken ends and generation of 3′ ssDNA tails, which are both an intermediate for DSB repair by TMEJ and HR, and a barrier for DSB repair by NHEJ (24,31–35). Resection is initiated by the MRN complex (Mre11, Rad50 and Nbs1) (Figure 4A) (32,36). We used two independent *Mre11^ATLD1^* MEF cell lines that have bi-allelic knock-in of a hypomorphic Mre11 allele (37–39) to assess the effects of these alleles on pathway choice using PathSig-dPCR. As expected, we confirmed significantly reduced protein levels of Mre11, Rad50, and Nbs1 in both *Mre11^ATLD1^* MEF lines by western blot, consistent with previously reported destabilization of the MRN complex induced by the *Mre11^ATLD1^* allele (Figure 4B) (39). After transfection with Cas9 and sgRosa26, we observed significant reductions in TMEJ-del 23bp and TMEJ-del 39bp in *Mre11^ATLD1^*-1 and *Mre11^ATLD1^*-2 cell lines, relative to *WT* MEFs (Figure 4C, D). We also found reduced frequency of HR repair in *Mre11^ATLD1^*-1 and *Mre11^ATLD1^*-2 cell lines upon co-transfection with the HRD-500 (Figure 4E). In contrast, the NHEJ-ins +1 bp product was slightly increased, although this difference did not achieve statistical significance (Figure 4F). Thus, PathSig-dPCR establishes essential roles for Mre11 in HR and TMEJ, but not in NHEJ, during chromosomal DSB repair in mammalian cells.

**Figure 5.**
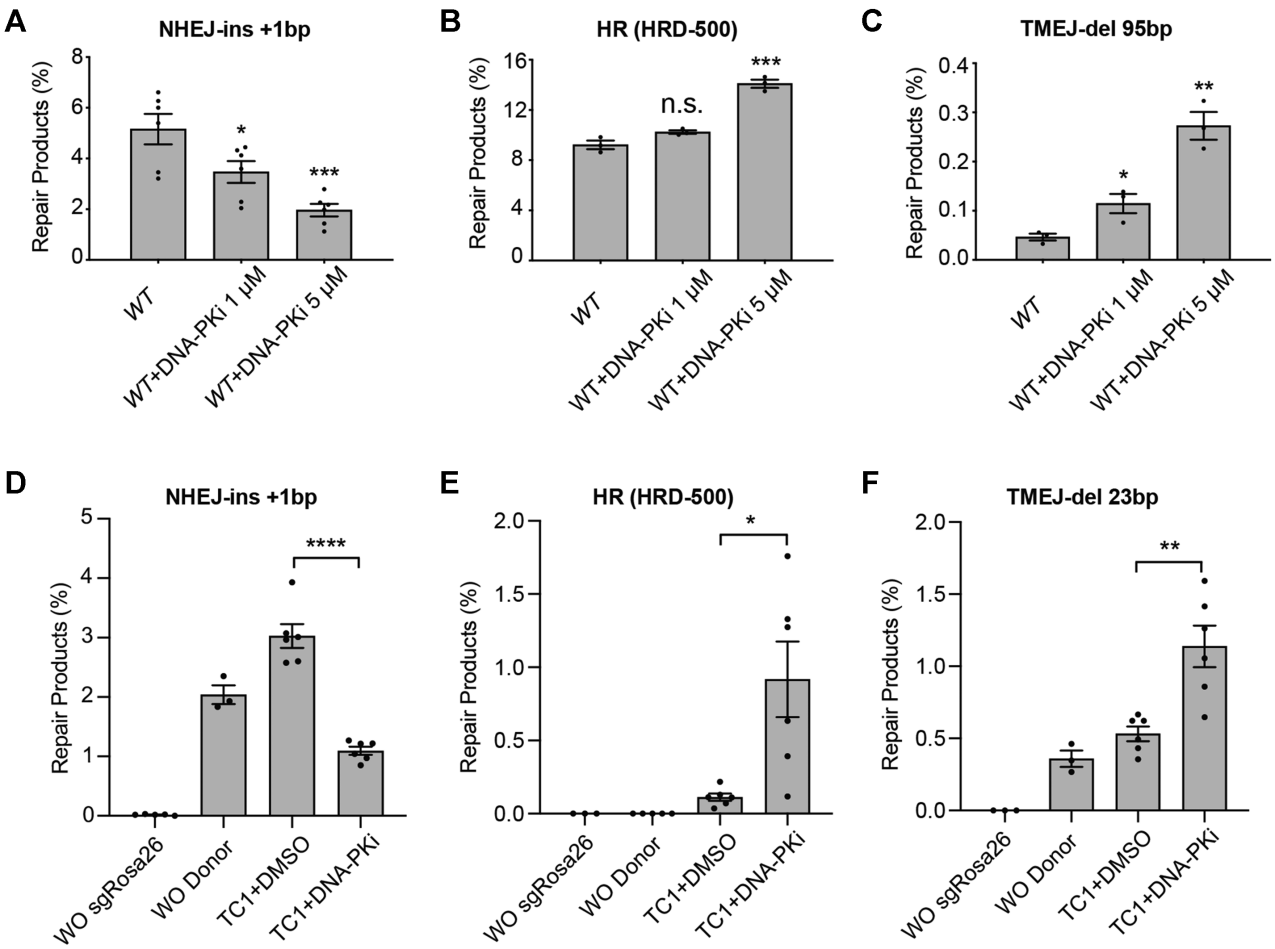
Inhibition DNA-PK alters DSB repair pathway utilization. (**A-C**) Evaluation of (**A**) NHEJ-Ins +1bp, (**B**) HR (HRD-500), and (**C**) TMEJ-del 95bp signature repair products after increasing doses of DNA-PKi in *WT* MEF cells. (D–F) ES cells (TC1) were transfected with Cas9-RNP targeting the Rosa26 locus and a homologous donor (HRD-500), followed by PathSig-dPCR assays for (**D**) NHEJ-Ins +1 bp, (**E**) HR repair (HRD-500), and (**F**) TMEJ-del 23 bp. Mean ± SEM are shown. Statistical significance was assessed by two-tailed *t*-tests. * *P* < 0.05, ** *P* < 0.01, *** *P* < 0.001 and **** *P* < 0.0001.

Pharmacologic inhibition of DNA-PK (DNA-PKi) has been shown to inhibit NHEJ and may increase the efficiency of HR and TMEJ (40). We used PathSig-dPCR to quantify these effects using different doses of the DNA-PKi Nu7441. We observed ∼33% reduction in NHEJ repair in response to 1 μM DNA-PKi, and 67% reduction at 5 μM Nu7441 (Figure 5A). HR was also increased upon treatment with DNA-PKi, resulting in a 50% greater efficiency of gene conversion at the highest dose (Figure 5B). TMEJ repair products were also increased at the higher DNA-PKi dose, although modest increases in TMEJ were also seen at the lower DNA-PKi dose (Figure 5C, Supplementary Figure S7A,B). As anticipated, DNA-PKi treatment does not change DSB outcomes in *Ku70^−^^/^^−^* MEFs, which are already deficient in NHEJ (Supplementary Figure S7C–E).

A distinct advantage of the reporter-free PathSig-dPCR assay is its applicability to primary cell models. We thus applied PathSig-dPCR to a primary murine ES cell line (TC1) by co-transfecting Cas9, sgRosa26 and HRD-500 plasmids with or without DNA-PKi (5 μM Nu7441) treatment. NHEJ repair was reduced from 3% with no treatment to 1% with DNA-PKi treatment, while the usage of HR and TMEJ-del 23 bp were elevated from 0.1% to 0.9% and from 0.5% to 1.1%, respectively (Figure 5D–F). Lower rates of DSB repair products in TC1 cells can be attributed to a low transfection efficiency (<5%, Supplementary Figure S7F). After normalization for transfection efficiency, the gene conversion rate in TC1 cells is ∼20% in the presence of 5μM DNA-PKi. Our findings suggest that DNA-PK inhibition may be more impactful in stimulating HR when transfection efficiency is limited.

We further validated PathSig-dPCR by evaluating concordance with fluorescence-based DSB repair reporter assays (14,24,25). We first used a MEF cell line with an integrated DR-GFP reporter assay (24) to evaluate the effect of pharmacologic DNA-PK inhibition and CRISPR/Cas9 targeting of Rad51 on the efficiency of HR repair. PathSig-dPCR is highly concordant with DR-GFP flow cytometry under both of these experimental perturbations (Supplementary Figure S8). PathSig-dPCR further enabled quantification of NHEJ and TMEJ repair, revealing an increase in TMEJ upon both DNA-PK or Rad51 inhibition (Supplementary Figure S8). We also generated a MEF cell line stably expressing the Traffic Light Reporter (TLR), and evaluated changes in HR and NHEJ utilization upon treatment with a DNA-PK inhibitor (25). We observed high concordance between the fluorescent TLR readouts and PathSig-dPCR for HR and NHEJ (Supplementary Figure S9). PathSig-dPCR additionally demonstrated increased prevalence of TMEJ repair upon DNA-PK inhibition in this TLR-labeled cell population. Thus, PathSig-dPCR is highly concordant with fluorescence DSB reporter assays, and allows for parallel quantification of all three major DSB repair pathways in unlabeled cell populations.

### Kinetics of DNA repair in response to Cas9 induced DSB

**Figure 6.**
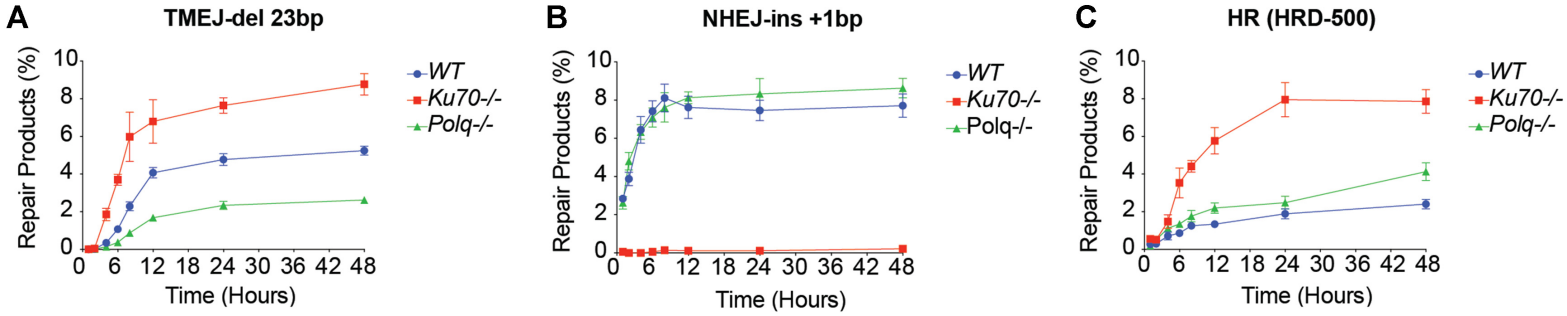
Kinetics of DSB repair in *WT*, *Ku70^−^^/^^−^* and *Polq^−^^/^^−^* cells. *WT, Ku70^−^^/^^−^* and *Polq^−^^/-^* MEFs were transfected with Cas9 ribonucleoprotein (Cas9-RNP) targeting the Rosa26 locus and a homologous donor (HRD-500). Cells were collected and extracted for genomic DNA at 1, 2, 4, 6, 8, 12, 24 and 48 h, and PathSig-dPCR assays were performed to detect NHEJ-ins +1 bp (**A**), HR repair (**B**), and TMEJ-del 23 bp (**C**). Error bars indicate ± SEM.

NHEJ is a rapid repair process, and HR is known to be relatively slower (41,42). While MMEJ/TMEJ has previously been shown to be slower than NHEJ (8), its kinetics relative to HR remain unknown. We implemented PathSig-dPCR in *WT*, *Ku70^−^^/^^−^* and *Polq^−^^/-^* MEFs to measure kinetics of NHEJ, HR, and TMEJ at a site-specific chromosomal DSB. Cells were transfected with a pre-assembled RNP-sgRosa26 complex (with or without HRD-500) to rapidly and synchronously induce DSBs, and Pathsig-dPCR used to monitor the accumulation of NHEJ, HR, and TMEJ repair products over time. In *WT* and *Polq^−^^/-^* cells, NHEJ repair products were rapidly detected as early as 1 hour post-transfection and peaked by 6 hours, with a time to 50% of maximum (*t*_1/2_) ∼ 2 hours (Figure 6A). In contrast, HR and TMEJ repair in *WT* cells had slower kinetics, with t_1/2_ ∼ 8 hrs for both (Figure 6B, C). DSB repair outcomes were substantially altered in *Ku70^−^^/^^−^* cells. HR-mediated repair was 3-fold more abundant and accumulated slightly more rapidly (*t*_1/2_ ∼ 7 h) than in *WT* cells (Figure 6B). Similarly, TMEJ was ∼80% more abundant with *t*_1/2_ ∼ 7 h (Figure 6C). As anticipated, NHEJ repair remained undetectable throughout the time course in *Ku70^−^^/^^−^* cells (Figure 6A). *Polq^−^^/-^* cells shared similar kinetics of NHEJ and HR repair, while HR repair in *Polq^−^^/-^* cells was ∼1.7 times more than in *WT* cells. As expected, TMEJ-like products were reduced in *Polq^−^^/^^−^* cells. Thus, implementation of PathSig-dPCR reveals that TMEJ exhibits comparable kinetics to HR—despite potentially requiring less extensive end resection—and both pathways are antagonized by Ku70.

## DISCUSSION

Accurate measurement of repair outcomes at Cas9-targeted DSBs is critical for investigating DSB repair mechanisms and for optimizing genome engineering. The methods described herein—ScarMapper and PathSig-dPCR—enable comprehensive and quantitative evaluation of HR, NHEJ, and TMEJ repair outcomes at any Cas9-targeted site in cellular models, and further provides validated primers for such analyses in mouse cell lines. The versatility of these assay platforms for any chromosomal locus of interest will enable the evaluation of DSB repair pathway choice in diverse experimental settings, including in primary cell models.

ScarMapper is a Python-encoded pipeline that facilitates scalable and automated classification of DSB repair outcomes, and represents a significant improvement over previously available algorithms (16,18,19). ScarMapper uses an iterative 10-mer matching algorithm for break site-specific alignment to precisely identify deletions, insertions, and microhomology usage for each of the sequenced repair products. An important advantage of ScarMapper is its ability to classify complex repair products that contain both inserted and deleted nucleotides more consistently than alignment-based algorithms (16,18). Graphical visualization of ScarMapper's tabular output reveals how the spectra of repair products is differentially affected by deficiency in *Ku70* or *Polq*, thus defining the dependency of these products on NHEJ versus TMEJ. ScarMapper can also be used for quantification of Pol θ dependence of any specific repair product, which may facilitate more systematic characterization of TMEJ repair at any locus of interest. Thus, ScarMapper enables comprehensive and automated analyses of chromosomal DSB repair outcomes using amplicon NGS.

A drawback of amplicon NGS for quantifying repair outcomes is its limit to quantifying relative levels of different classes of amplifiable products, without consideration of sample-dependent variation in the ability to amplify the locus (i.e. due to persistent breakage, or deletion of the binding sites for amplifying primers). PathSig-dPCR overcomes this barrier by enabling absolute quantification of specific repair products of interest relative to a distal genomic reference site. By designing allele-specific assays for specific ‘signature’ products identified using ScarMapper we illustrate how PathSig-dPCR can be used to robustly measure NHEJ, HR, and TMEJ repair efficiency in diverse experimental settings, such as upon manipulation of DSB end resection pathways. Unlike reporter-based repair assays, PathSig-dPCR can be applied to any unlabeled cell type, including primary or immortalized cells from transgenic mouse strains of interest.

Prior studies have demonstrated the utility of dPCR to probe kinetics of breakage, resection, and repair after the introduction of a targeted DSB by Cas9 (8,43). Here we extend such analysis to include ‘signature product’-based assessments of the relative contribution of the three different major DSB repair pathways, using genetic models and Scarmapper analysis of amplicon sequencing to rigorously validate the specificity of these signature products. We illustrate how PathSig-dPCR assays can be implemented in a cost- and labor-efficient manner, revealing extremely rapid kinetics of NHEJ repair—detectable within 30 minutes and largely completed by 6 h post-transfection. In contrast, TMEJ and HR exhibit slower kinetics, and saturate by about 24 h. *Ku70* deficiency increases both the frequency and kinetics of HR and TMEJ repair. We anticipate PathSig-dPCR will facilitate further investigation of mechanisms that regulate the choice between NHEJ, HR, and TMEJ pathways, as well as their respective kinetics of repair.

ScarMapper and PathSig-dPCR are complementary assays and can be adapted to measure DSB repair pathway utilization at any chromosomal locus that can be targeted by Cas9. ScarMapper analysis of amplicon NGS characterizes the full spectrum of HR and end-joining repair that arises after Cas9-mediated cleavage, with automated repair product classification according to deletion, insertion, and MH size. PathSig-dPCR provides the complementary ability to absolutely quantify specific repair outcomes of interest, without requiring pathway-specific reporter constructs. The combination of these highly tractable assays will facilitate future studies of DSB repair pathway choice and optimization of Cas9-mediated genome engineering.

## DATA AVAILABILITY

Further information and requests for resources and reagents should be directed to and will be fulfilled by the Lead Contact, Gaorav Gupta (gaorav_gupta@med.unc.edu). The ScarMapper pipeline is publicly available through GitHub (https://github.com/Gaorav-Gupta-Lab/ScarMapper.git). Next-generation sequencing data will be made available in the Sequence Read Archive (SRR13044016). Data analysis files for each figure will be made available through MendeleyData (https://data.mendeley.com/datasets/3nw4bztjns/1). All unique/stable reagents generated in this study are available from the Lead Contact with a completed Materials Transfer Agreement.

## Supplementary Material

gkab299_Supplemental_Files
