# Supplementary material for: Marker-free quantification of repair pathway utilization at Cas9-induced double-strand breaks
Source: Nucleic Acids Res. 2021 May 8;49(9):5095–105. doi: 10.1093/nar/gkab299 (PMC8136827; doi:10.1093/nar/gkab299)
Supplement: gkab299_Supplemental_Files [file gkab299_supplemental_files.zip › SupplementaryFigures_v3.docx]

**Supplementary Figures and Legends**

**
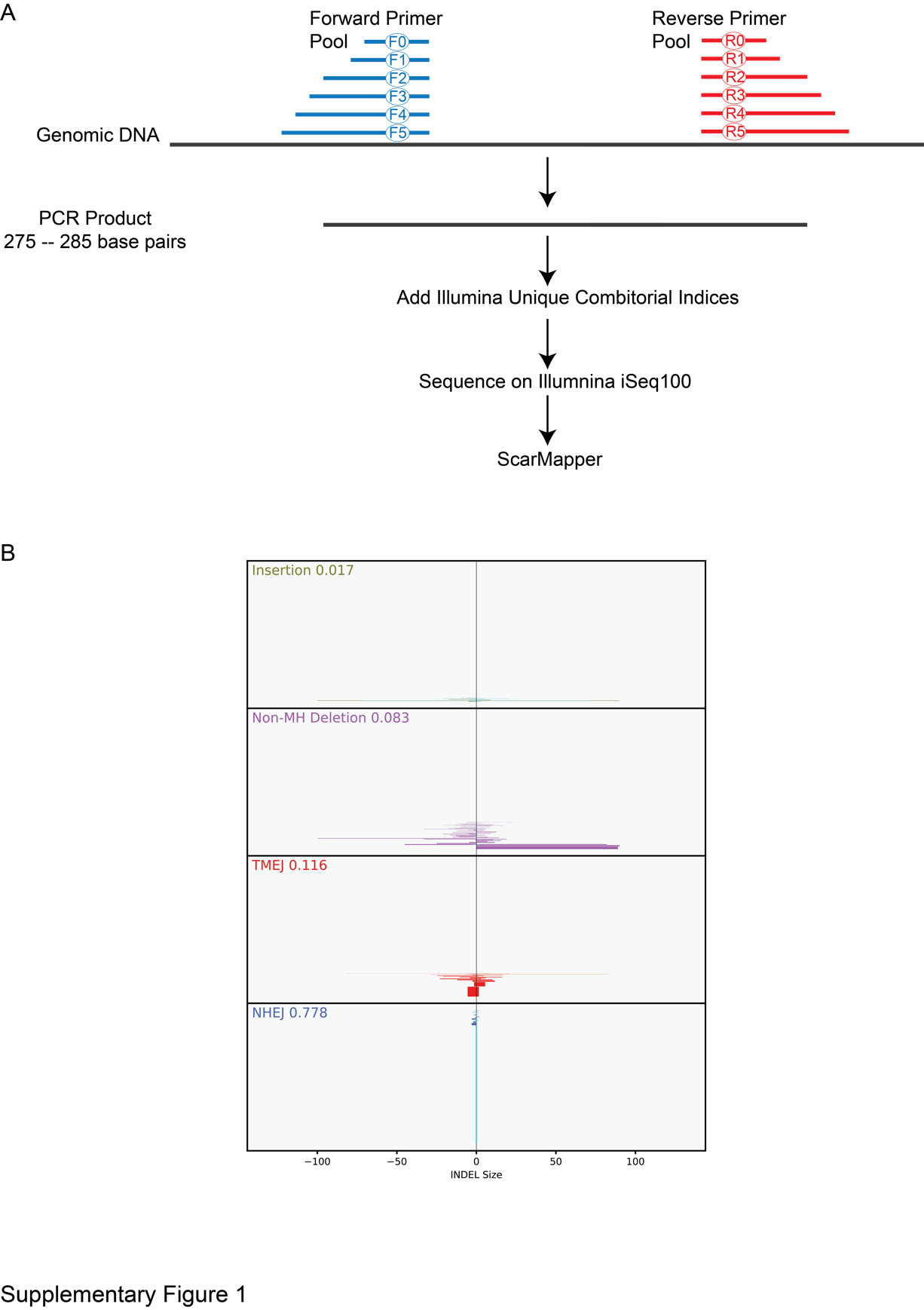
**

**Supplementary Figure 1:** ScarMapper Analysis on Human Lamin B Receptor (*LBR*) Locus in hTERT-RPE1 Cells. (A) Schema showing the library prep method. A phased primer strategy is used for first round PCR amplification, followed by a second round PCR to add Illumina adaptors and unique dual indices. (B) Plot of ScarMapper output results.

**
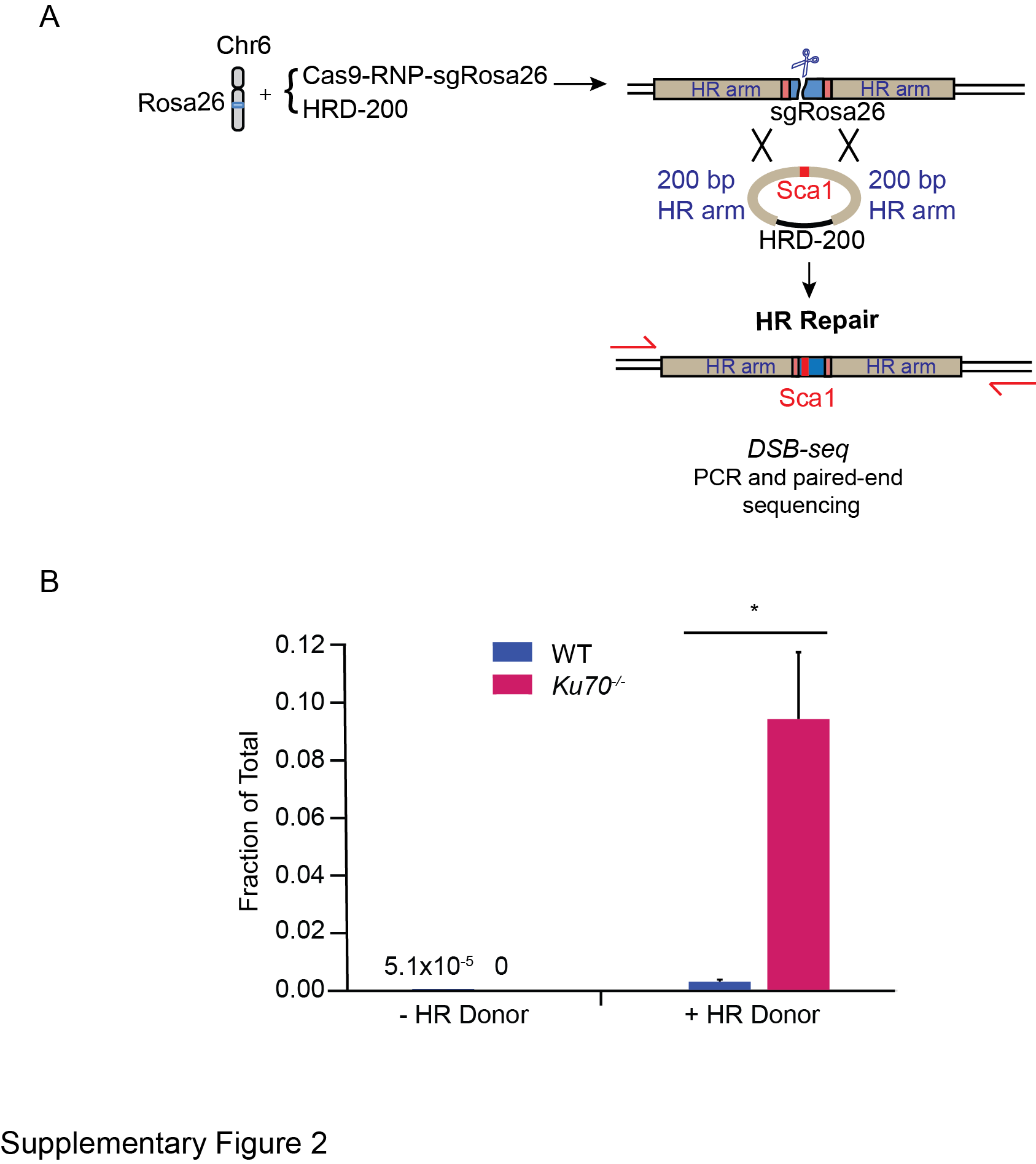
**

**Supplementary Figure 2:** ScarMapper Analysis on HR repair product in *WT* and *Ku70^-/-^* MEFs. (A) sgRNA targeting Rosa 26 locus was transfected into transformed mouse embryonic fibroblasts (MEFs) along with Cas9 protein. HRD-200 was co-transfected with Cas9-RNA-sgRosa26. 48 hours after transfection the cells are harvested, genomic DNA is purified, the target region is PCR amplified from the genomic DNA followed by sequencing. (B) Histogram showing homologous recombination repair measurements in *WT* and *Ku70^-/-^* MEFs. Shown are mean +/- SEM. Significance by two-tailed Student’s t-test. P-value=0.0118.

**
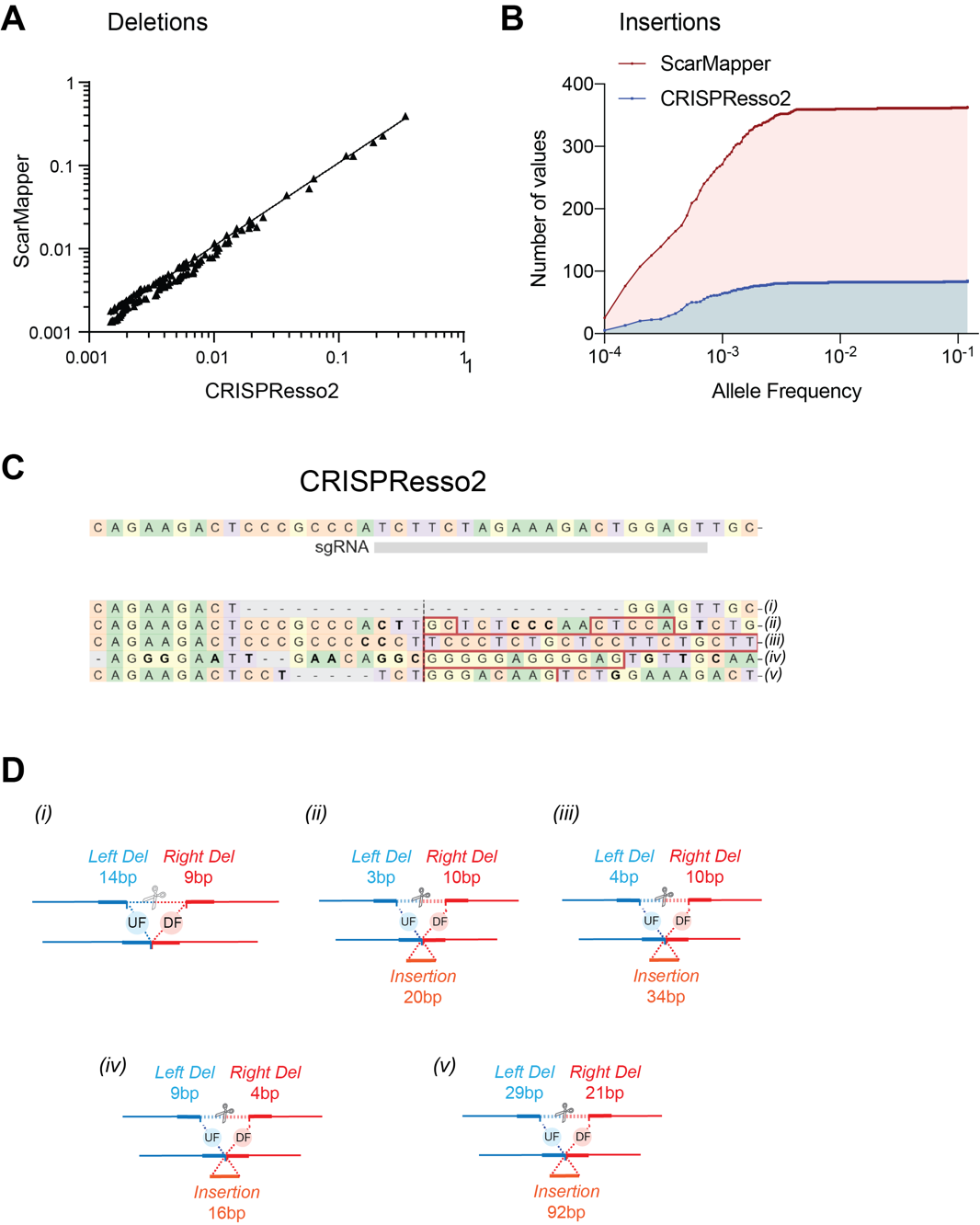
**

**Supplementary Figure 3:** Performance comparison of ScarMapper and CRISPResso2. (A) Scatter plot comparing the frequency of the top 50 deletion patterns in ScarMapper to those derived from CRISPResso2. Both programs identify and score global deletions accurately. (B) Cumulative frequency plot of the global insertions with or without deletions identified by ScarMapper and CRISPResso2 in amplicon NGS data from *Ku70^-/-^* MEFs. ScarMapper identifies 363 insertions, whereas CRISPResso2 identifies 85. (C) CRISPResso2-based alignment of five illustrative repair products, (*i) - (v)*, obtained from NGS analysis of *Ku70^-/-^* MEFs. Bold nucleotides are scored as substitutions, dashes represent deletions and nucleotides in the red boxes are insertions. The cleavage site is shown as a dashed line. (D) Same 5 patterns shown in (C) as scored by ScarMapper, revealing clear demarcations of deleted and inserted nucleotides.

**
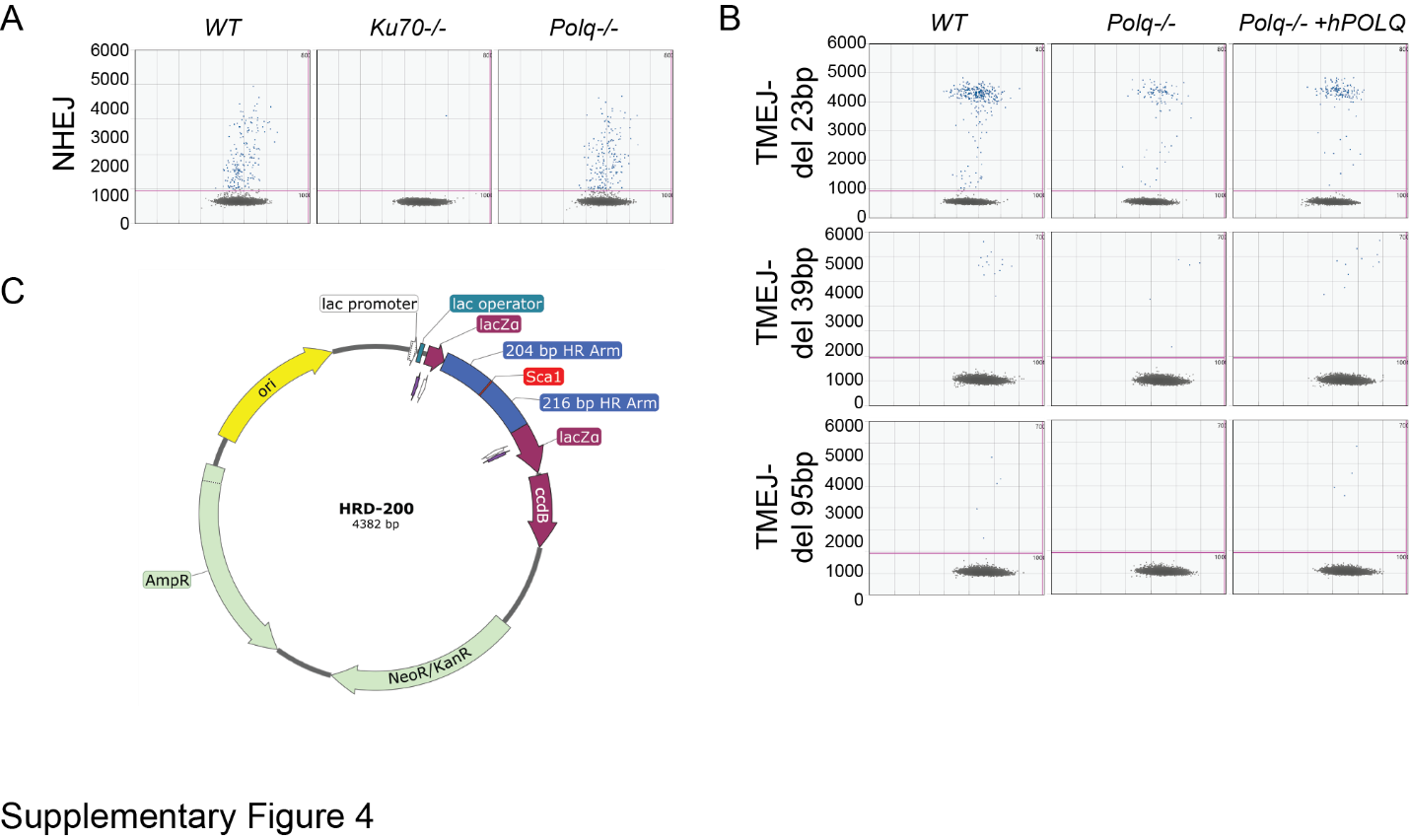
**

**Supplementary Figure 4:** Additional details of the PathSig-dPCR assays for NHEJ, TMEJ and HR signature products. (A) Representative dPCR dot plots for detection of NHEJ-Ins +1bp repair product in *WT*, *Ku70-/-* and *Polq-/-* cells. (B) Representative dPCR dot plots for detection of TMEJ-del 23bp, TMEJ-del 39bp and TMEJ-del 95bp repair products in *WT*, *Polq-/-* and *Polq-/-* +hPOLQ cells. (C) Map of HR 200 bp donor. 216 bp of identity to sequence upstream of the Rosa26 target, a central segment sufficient to destroy recognition by the Rosa26 targeting guide and creation of a ScaI restriction enzyme site, followed by 204 bp of identity to downstream sequence, cloned into pCR4 Topo cloning vector.

**
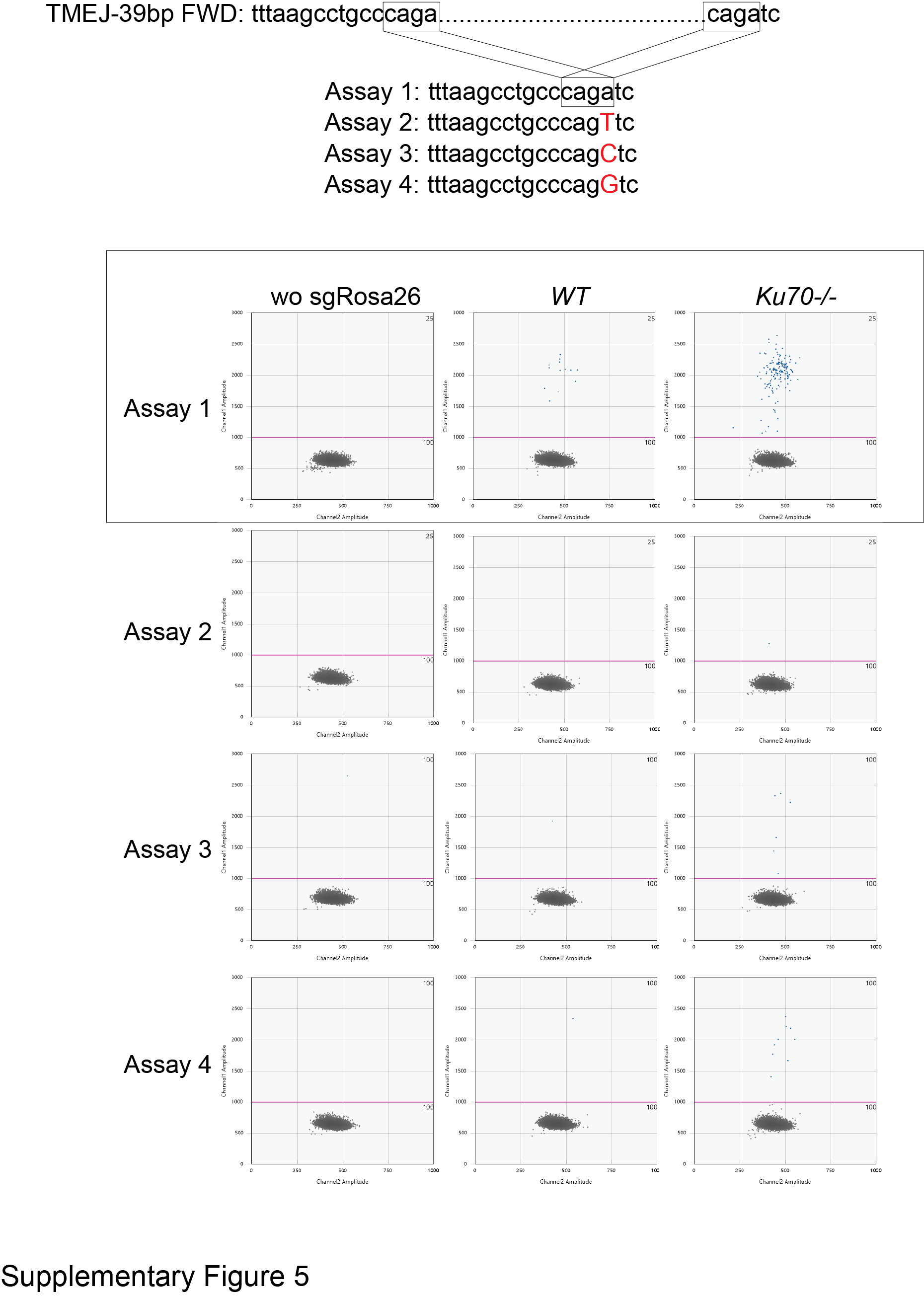
**

**Supplementary Figure 5:** Design of allele-specific forward primer for TMEJ-del 39bp repair product. TMEJ-del 39bp repair product has 39 base pair deletion with the use of 4 base pair microhomology “CAGA”. TMEJ-39bp FWD spans across the junction and primers with or without mismatches to destabilize the primer against amplification of the wild-type sequence were evaluated by dPCR. Assay 1 exhibited the greatest sensitivity and specificity for the TMEJ-del 39bp repair product.


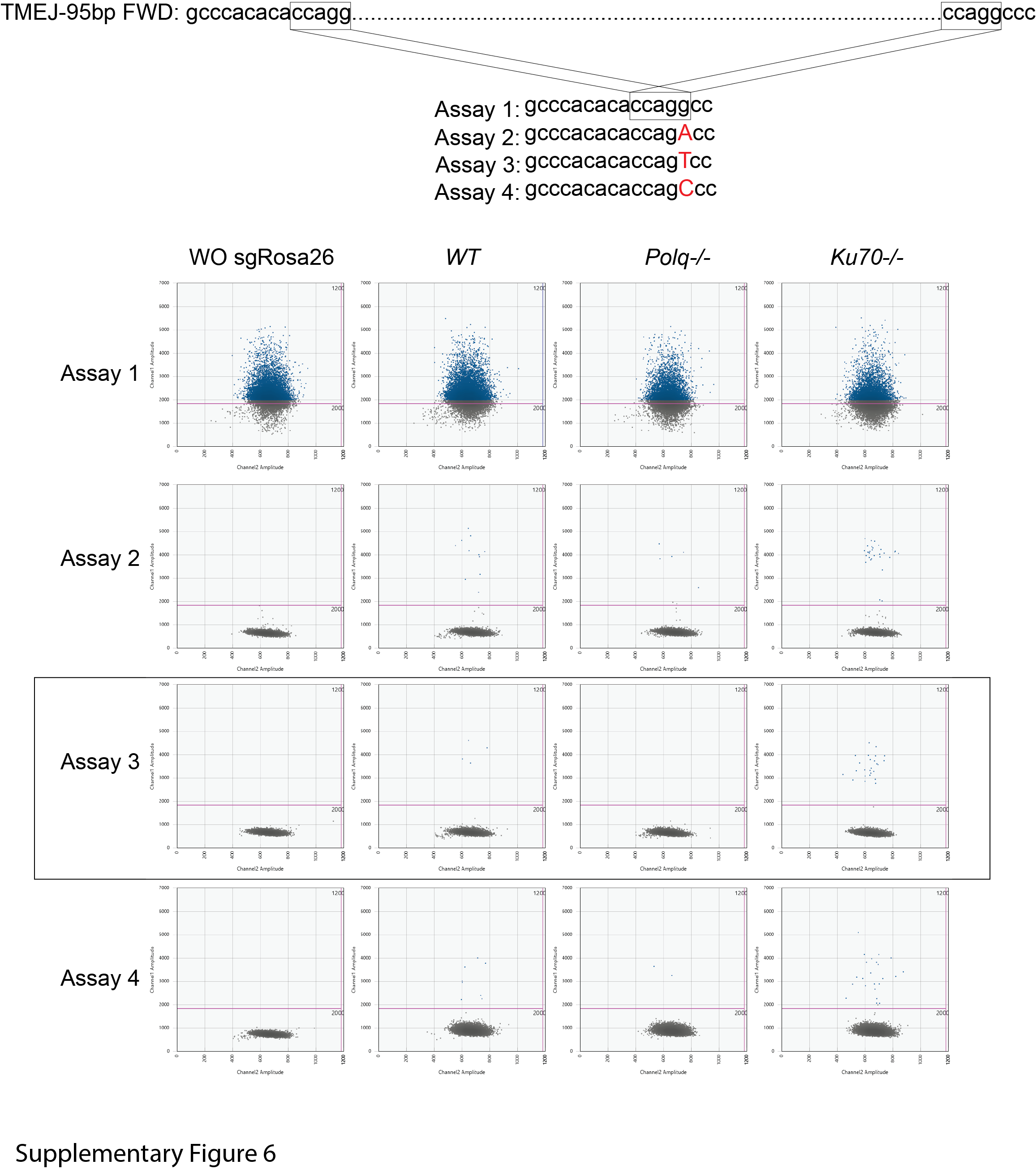


**Supplementary Figure 6:** Design of allele specific forward primer for TMEJ-del 95bp repair product. TMEJ-del 95bp repair product has 95 base pair deletion with the use of 5 base pair microhomology “CCAGG”. TMEJ-95bp FWD spans across the junction and primers with or without mismatches to destabilize the primer against amplification of the wild-type sequence were evaluated by dPCR. Assay 3 exhibited the greatest sensitivity and specificity for the TMEJ-del 95bp repair product.


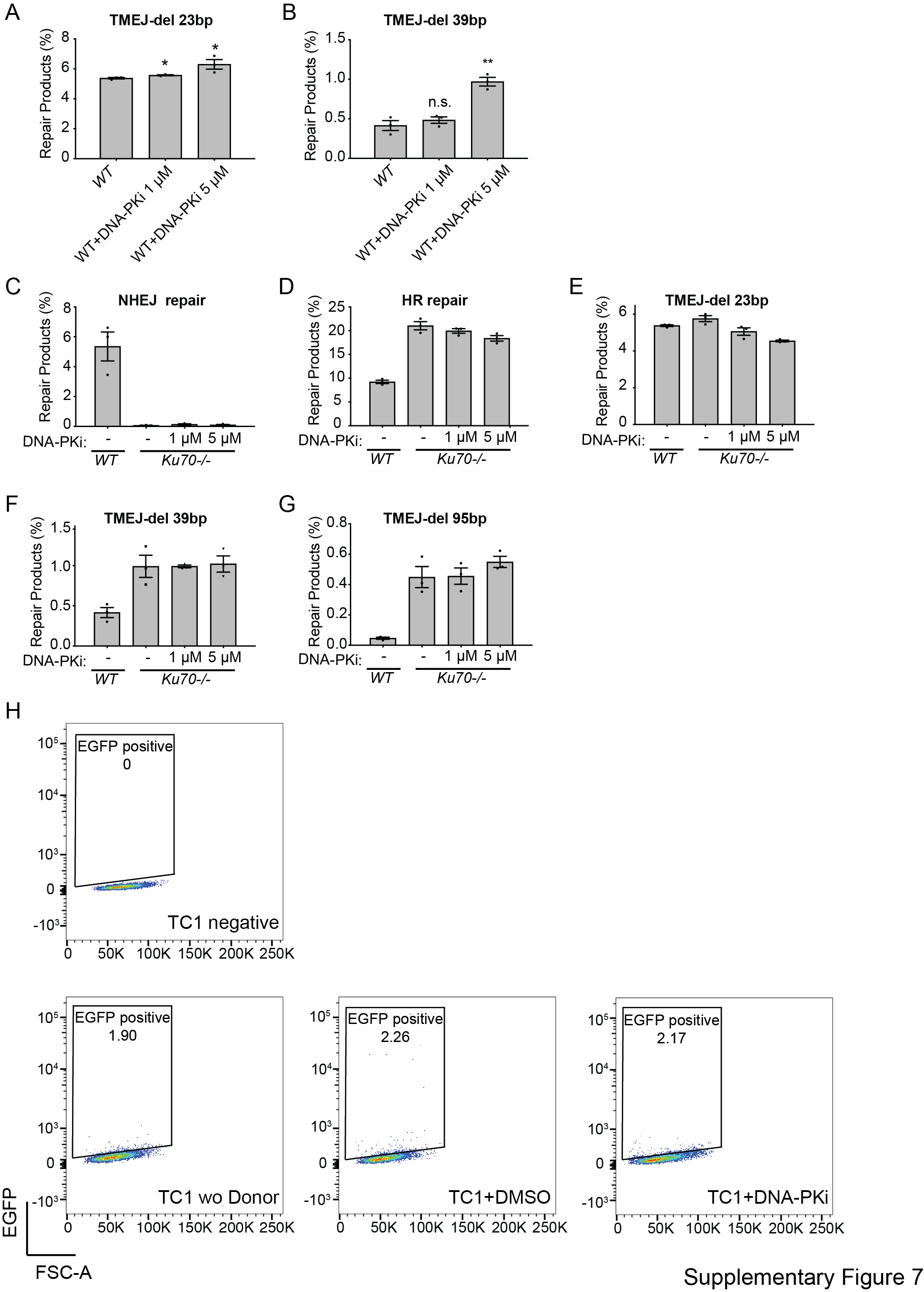


**Supplementary Figure 7**: Explore DNA repair choice in response to DNA-PKi in *Ku70-/-* cells. (A) Very low level of NHEJ product was detected in *Ku70-/-* cells with or without DNA-PKi. No significant change was observed in HR repair (B), TMEJ-del 23 bp (C), TMEJ-del 39 bp (D) and TMEJ-del 95 bp (E) in response to different doses of DNA-PKi in *Ku70-/-* cells. (F) Transfection efficiency was measured by percentage of EGFP positive cells detected by flow cytometry in ES (TC1) cells. Plasmids with expression of Cas9, sgRosa26, with or without HR donor and EGFP were transfected into ES (TC1) cells. ~2% cells were EGFP positive cells. ES (TC1) without any transfection were used as a negative control.


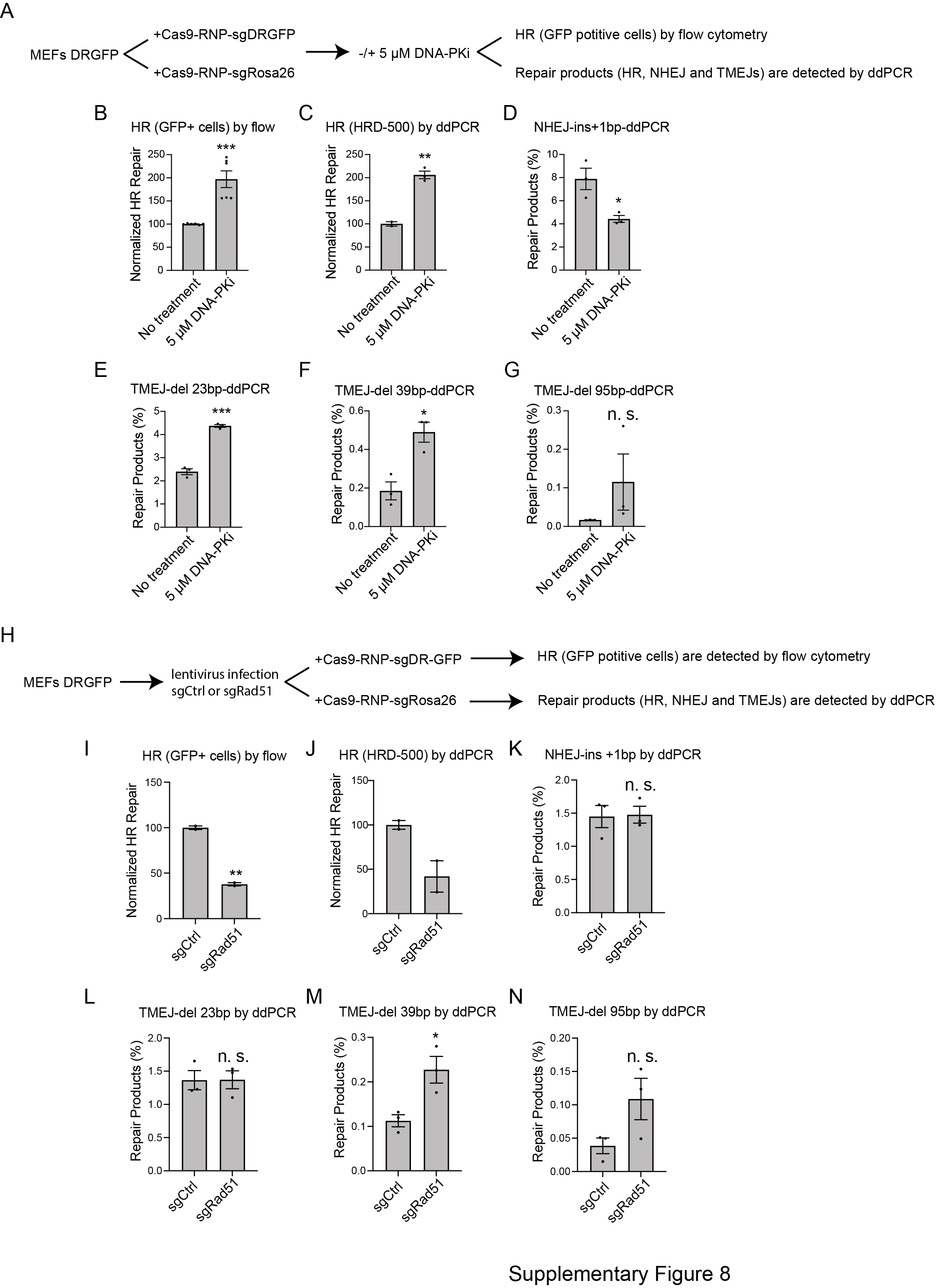


**Supplementary Figure 8:** Compare DR GFP reporter assay with PathSig-dPCR. (A) MEFs DRGFP were transduced with RNP-sgRosa26 or RNP-sgDRGFP by Neon with or without DNA-PKi. (B) HR events were presented by GFP positive cells detected by flow. HR events were normalized to untreated cells. (C-G) HR, NHEJ-ins+1bp, TMEJ-del 23bp, TMEJ-del 39bp and TMEJ-del95 were detected by PathSig-dPCR. (H) MEFs DRGFP were CRISPR-targeted for HR factor Rad51. HR reduced dramatically in sgRad51 target cells compared to control sgRNA cells in both of DRGFP assay (I) and PathSig-dPCR (J). (K-N) NHEJ-ins+1bp, TMEJ-del 23bp, TMEJ-del 39bp and TMEJ-del 95bp were detected by PathSig-dPCR.


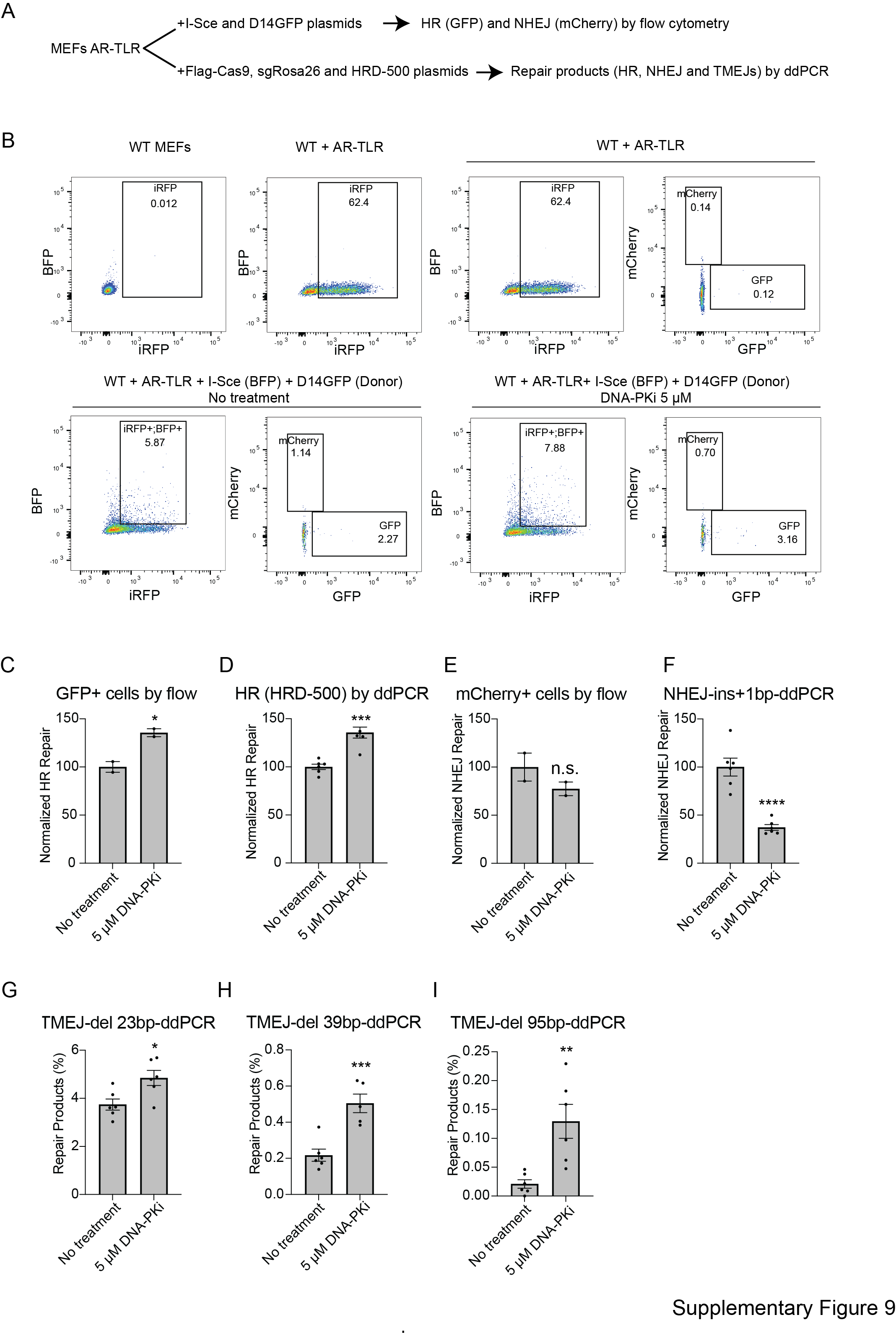


**Supplementary Figure 9:** Compare Traffic Light Reporter (TLR) assay with PathSig-dPCR. (A) MEFs AR-TLR were transduced with Flag-Cas9, sgRosa26 and HRD-500 for PathSig-dPCR or transfected with I-Sce and D14GFP for TLR assay in present/absent of DNA-PKi. (B) Flow cytometric analysis of MEFs AT-TLR 48 hours after transfection. BFP expression is a marker for transfection efficiency. (C) HR events were presented by GFP positive cells detected by flow. HR events were normalized to untreated cells. (E) NHEJ events were presented by mCherry positive cells detected by flow. NHEJ events were normalized to untreated cells. HR (D), NHEJ-ins+1bp (F), TMEJ-del 23bp (G), TMEJ-del 39bp (H) and TMEJ-del95 (I) were detected by PathSig-dPCR.
