# Supplementary material for: Marker-free quantification of repair pathway utilization at Cas9-induced double-strand breaks
Source: Nucleic Acids Res. 2021 May 8;49(9):5095–105. doi: 10.1093/nar/gkab299 (PMC8136827; doi:10.1093/nar/gkab299)
Supplement: gkab299_Supplemental_Files [file gkab299_supplemental_files.zip › Suppmentary Methods.docx]

**Part 1: ScarMapper User Guide;**

**Part 2: PCR Conditions;**

**Part 3: Step by step ddPCR.**

**Part 1: ScarMapper User Guide:**

# ScarMapper User Guide

ScarMapper is a pipeline for identification and analysis of repair products at targeted double strand breaks by next generation sequencing. The primer phasing part of this pipeline consists of best practices to design the phased primers required to successfully sequence the amplicons on Illumina platforms. The repair scar search function consists of a Python-encoded algorithm that uses an iterative break-associated alignment strategy to classify individual double-strand DNA break repair products based on deletion size, microhomology usage, and insertions. This method has advantages over other methods such as CRISPResso2, RIMA, and HiFiBR [1-3] in ease of use, ability to classify complex insertions, speed and flexibility in creating the consensus sequences. Furthermore, ScarMapper can simultaneously analyze multiple loci within a single sample.

## Primer Phasing

Depending on how the primers were designed, these sequencing libraries will contain no diversity for the first 30 – 50 nucleotides. To solve this, primers used to create the amplicon for sequencing need to be phased. NGS_PrimerPhasing (<https://github.com/Gaorav-Gupta-Lab/NGS_PrimerPhasing.git>) can be used to assist in designing phased primers. Depending on the template, a pool of 4 – 6 forward and reverse phased primers are required. For example, primers we have used for the Lamin Receptor B locus.

| **Primer Name** | **Orientation** | **Location** | **Sequence** |
| --- | --- | --- | --- |
| LBR2.1 F0 | Forward | chr1:225423928-225423949 | CGACGCTCTTCCGATCTTCAATTCAAGCTCTGTTCCATC |
| LBR2.1 F1 | Forward | chr1:225423927-225423949 | CGACGCTCTTCCGATCTTTCAATTCAAGCTCTGTTCCATC |
| LBR2.1 F2 | Forward | chr1:225423927-225423949 | CGACGCTCTTCCGATCTCTTCAATTCAAGCTCTGTTCCATC |
| LBR2.1 F3 | Forward | chr1:225423927-225423949 | CGACGCTCTTCCGATCTACTTCAATTCAAGCTCTGTTCCATC |
| LBR2.1 F4 | Forward | chr1:225423927-225423949 | CGACGCTCTTCCGATCTGACTTCAATTCAAGCTCTGTTCCATC |
| LBR2.1 F5 | Forward | chr1:225423927-225423949 | CGACGCTCTTCCGATCTAGACTTCAATTCAAGCTCTGTTCCATC |
| LBR2.1 R0 | Rcomp | chr1:225424162-225424143 | CGTGTGCTCTTCCGATCTTCAGCCTGTGGAAAAAGACG |
| LBR2.1 R1 | Rcomp | chr1:225424163-225424143 | CGTGTGCTCTTCCGATCTATCAGCCTGTGGAAAAAGACG |
| LBR2.1 R2 | Rcomp | chr1:225424164-225424143 | CGTGTGCTCTTCCGATCTGATCAGCCTGTGGAAAAAGACG |
| LBR2.1 R3 | Rcomp | chr1:225424165-225424143 | CGTGTGCTCTTCCGATCTTGATCAGCCTGTGGAAAAAGACG |
| LBR2.1 R4 | Rcomp | chr1:225424166-225424143 | CGTGTGCTCTTCCGATCTCTGATCAGCCTGTGGAAAAAGACG |
| LBR2.1 R5 | Rcomp | chr1:225424167-225424143 | CGTGTGCTCTTCCGATCTACTGATCAGCCTGTGGAAAAAGACG |

The sequence that matches the target is TCAATTCAAGCTCTGTTCCATC. The sequence that is not highlighted is for adding the Illumina indices. The phasing sequence is highlighted in green (CT). Phase 0 primers, F0 or R0, contain no extra nucleotides.

## Prerequisites for running ScarMapper

1. Linux OS, tested on RHEL 7.x, Scientific Linux 7.x, and CentOS 7.x. Will possibly run on a Mac OS, although it has not been tested. Will not run on Windows.
2. Ideally ≥32 Gb RAM. The amount of RAM depends on the number of reads being processed. This amount will allow processing of a FASTQ file containing ~15 million read pairs. The minimum recommended amount of RAM is 24 Gb. This will allow processing of ~8 million read pairs. The RAM does not scale equally with the number of reads.
3. >2 CPUs or threads. Processes one library per CPU/thread in parallel. The number of parallel jobs possible is CPUs or threads minus 1.
4. The minimum available drive space required is approximately 500 Mb. This does not include the space required for the FASTQ files.
5. Python ≥3.5
   1. Python ≥3.6 recommended
6. Python packages, latest version of each
   1. scipy
   2. natsort
   3. matplotlib
   4. pysam
   5. python-magic
   6. pathos
   7. pandas
   8. numpy
   9. python-Levenshtein
   10. cython
   11. setuptools

## Installation

1. Download or clone ScarMapper from GitHub (https://github.com/Gaorav-Gupta-Lab/ScarMapper.git) to a location you have read/write access.
2. Test installation by moving to the ScarMapper directory and executing <python3 scarmapper.py> (without the <>). You should get the error message below.
   1. usage: scarmapper.py [-h] --options_file OPTIONS_FILE
       scarmapper.py: error: the following arguments are required: --options_file

## Required Files

ScarMapper requires the files listed below. Examples of the Master Index file and the Targets file can be found in the docs folder of ScarMapper. The formats and contents of each of these is discussed later.

1. Genome Reference Sequence in FASTA format.
   1. Obtain a reference genome of your choice such as GRCh38 or GRCm38. Make sure you also have the .fai index file. If one was not available to download you will need to create it with Samtools. Place these files in a directory you have access to.
2. Sample Manifest File
   1. Tab delimited text file. The excel template (ScarMapper_Sample_Manifest.xlsx) is found in the docs folder. As a minimum, columns A through G are required for ScarMapper. Either copy and paste those columns into a text editor or export the sheet from the excel file as a tab delimited csv file. The rows that begin with a # are treated as a comment line.
3. Paired end FASTQ files.
   1. Place these somewhere you have read access to. ScarMapper is designed to use compressed, paired end, multiplexed FASTQ files as input. Uncompressed files or a demultiplexed FASTQ file will also work.
4. Master Index File.
   1. This file is derived by copying the first three columns from the sheet in the **ScarMapper_Sample_Manifest.xlsx** file that corresponds to your sequencing platform and sequencing design. The file should be saved as a tab delimited text file. The table below shows the layout for Illumina dual indexing.

| **# Index_ID** | **Forward (D7s)** | **Reverse (D5s)** |
| --- | --- | --- |
| D501+D701 | ATTACTCG | TATAGCCT |

1. Targets File
   1. This is a tab delimited text file. File ScarMapper_Targets.csv, found in the docs folder, shows how the file is to be laid out. The first row must start with a #. Place this file in a location you have, at a minimum, read access. The same targeted region can contain multiple sgRNA sequences. These are treated as unique targets and go on individual lines. Each column is explained below.
   2. TargetName
      1. Unique name for the target. Avoid using special characters (`~!@#$%^&(*}{).
   3. Chr
      1. Chromosome target is on.
   4. Start
      1. 5’ genomic position of PCR amplicon containing cut site.
   5. Stop
      1. 3’ genomic position of PCR amplicon containing cut site.
   6. sgRNA_Seq
      1. The 5’ → 3’ sequence of the sgRNA. This is not relative to the genomic sequence. The cleavage site will always be at the -3 position from the 3’ end.
   7. ReverseComp
      1. If the sgRNA sequence is not on the forward strand of the genomic DNA then answer “Yes”, otherwise answer “No”.
   8. Comments
      1. Any notes or other words of wisdom about this target.
2. Options File
   1. This is a shell file that must be a formatted as a tab delimited text file. In the docs folder you will find two template options files; *run_ScarMapper_IndelProcessing.sh* for INDEL searching and *run_ScarMapper_Combine.sh* for combining output files.

## run_ScarMapper_IndelProcessing File

This option file is used to process the FASTQ files and find the INDELs. Each parameter is described below.

--IndelProcessing

Options are True or False. Must be True.

--FASTQ1

Full path to FASTQ1

--FASTQ2

Full path to FASTQ2

--RefSeq

Full path to the FASTA genomic reference. The reference index file must be in the same location as the genomic FASTA file

--Master_Index_File

Full path to the master index file described above.

-- SampleManifest

Full path to the pooled sample manifest file.

--TargetFile

Full path to the target file.

--WorkingFolder

Full path to a working folder. This is where ScarMapper will write the output files.

--Verbose

Set verbosity level. Options are INFO, DEBUG, ERROR. For general runs leave this at INFO. DEBUG will only process the first 1 million reads

--Job_Name

Provide a name for a run. White space is not allowed. This name will be incorporated into the output files simplifying identification. For this reason avoid special characters such as (`~!@#$%^&(*}{).

--Spawn

Defines how many parallel jobs to create. Each library defined in the sample manifest file will be processes in its own job. Max setting should be number of CPUs – 1. Minimum value is 1.

--Demultiplex

Allowed values are True or False. Defines if demultiplexed FASTQ files are written. If the files are written, they are automatically compressed with gzip with a compression setting of 9.

--HR_Donor

Sequence used for homologous recombination. If left blank no HR search is done. Recommend 10 – 12 nucleotides.

--Platform

Currently Illumina, or Ramsden. This tells ScarMapper how to identify the indices. Ramsden is a custom method that is in the publication.

--Minimum_Length

Minimum read length. Default is 100 nucleotides. This is depreciated and will be removed in future versions.

--OutputRawData

Allowed values are True or False. Determines if additional files are written. The raw data files are very large. They are currently not compressed. They contain the each read that has an identifiable repair scar. The data in these files are used to create the frequency output files.

# This next section is for PEAR. Any of these parameters can be left blank to use the default value.

--TestMethod

See PEAR documentation for how each method determines the consensus sequence.

--PValue

Used by PEAR to filter out bad consensus sequences. Default is 0.01.

--Memory

Defines memory reserved for PEAR. Default is 200 M, recommended value is >1000 M. There is a bug in PEAR such that the G flag (Gb) is not recognized.

--MinOverlap

Minimum overlap to generate the consensus sequence. The default is 10.

--QualityThreshold

Default is 40.

--PhredValue

Default is 33

--MinConsensusLength

Default value is 50.

# Ploting Options

--FigureType

Defines the output type of the waterfall plots. Allowed options are svg, jpg, tiff, pdf, and png. These are case sensitive.

## run_ScarMapper_Combine File

This module will combine the data in a group of frequency output files into a single file and draw a waterfall plot of the data.

--IndelProcessing False

Must be False.

--WorkingFolder /full/path/to/file/save/location/

This is where the output files will be written

--DataFiles /full/path/to/frequency/files/

This is the location of the data files to combine. ScarMapper will combine every data file present in the folder matching the name **ScarMapper_Frequency.txt*

--Verbose

Set verbosity level. Options are INFO, DEBUG, ERROR. For general runs leave this at INFO. DEBUG will report a small amount of additional information about the run.

--Job_Name

Provide a name for a run, it does not have to be the same as the names on the group of frequency files. White space is not allowed. This name will be incorporated into the output files simplifying identification. For this reason avoid special characters such as (`~!@#$%^&(*}{).

--SampleName

This will be part of the output file name and will be written in the waterfall plot.

# Plot Options

--FigureType

Defines the output type of the waterfall plots. Allowed options are svg, jpg, tiff, pdf, and png. These are case sensitive.

## Indel Processing Output Files

There are three primary and one optional output files from the INDEL processing module. The main file contains the summary data will be labeled Job_Name_Index_ScarMapper_Summary.txt. The next output file containing the scar data will be labeled Job_Name_Index_ScarMapper_Frequency.txt. The final primary output is a waterfall plot of the data in the format selected. There is a frequency file for each sample and a waterfall plot for each sample that has a scar fraction >10 %. The final output is optional and will contain the scar pattern found for each read. The Job_Name component is from the --Job_Name parameter described above. The “Index” component is from the first column in the -- SampleManifest. These files will be written in the location defined in the --WorkingFolder parameter. In all cases left and right is relative to the Cas9 cut site based on the sgRNA. If the sgRNA is reverse compliment as defined in the target file, then the sequence listed is the reverse compliment. The column contents are below.

ScarMapper Summary File

- ScarMapper 0.22.0
  - This is the first line in the file. It reports the program version.
- Start: Tue Mar 17 12:18:14 2020
  - This is the date and time the job was started.
- End: Tue Mar 17 12:21:58 2020
  - This is the date and time the job was completed.
- FASTQ1: /mnt/hgfs/Drive_D/Working/12-March-2020_R1.fastq.gz
  - This is FASTQ1
- FASTQ2: /mnt/hgfs/Drive_D/Working/12-March-2020_R2.fastq.gz
  - This is FASTQ2
- Reads Analyzed: 3591107
  - The number of reads analyzed. This is the number of consensus sequences from PEAR, not the number of reads in the FASTQ files.

# The next are the column headers.

- Index Name
  - From the sample manifest. The “Unidentified” index name is a collection of reads that do not match the sample manifest.
- Sample Name
  - From the sample manifest
- Sample Replicate
  - From the sample manifest.
- Target
  - Target locus from the sample manifest.
- Total Found
  - Number of reads with this index.
- Fraction Total
  - (Total Found) / (Reads Analyzed)
- Passing Read Filters
  - Total Found passing N and length filter
- Fraction Passing Filters
  - (Passing Read Filters) / (Reads Analyzed)
- No Read 1 Phasing
  - Read 1 sequences with no identifiable phasing. Usually due to a sequencing error.
- No Read 2 Phasing
  - Read 2 sequences with no identifiable phasing. Usually due to a sequencing error.

The number of columns for the phasing is automatically generated based on how many nucleotides were provided for the phasing sequence in the sample manifest.

- Phase F0
  - Fraction of reads that are forward phase 0.
- Phase F1
  - Fraction of reads that are forward phase 1.
- Phase F2
  - Fraction of reads that are forward phase 2.
- Phase R0
  - Fraction of reads that are reverse phase 0.
- Phase R1
  - Fraction of reads that are reverse phase 1.
- Phase R2
  - Fraction of reads that are reverse phase 2.
- No Junction
  - How many consensus sequences did not contain an identifiable junction?
- Scar Count
  - How many consensus sequences contained scars?
- Scar Fraction
  - (Scar Count) / (Passing Read Filters)

# If a HR sequence is given in the options file, then the HR columns will be present.

- HR Count
  - First number is the count of HR products seen once and second value is a count of how many products are seen more than once.
- HR Fraction
  - (sum(HR Count))/(Passing Read Filters)
- Left Deletion Count
  - How many consensus sequences contain a deletion to the left of the cut site with or without insertions or microhomology?
- Right Deletion Count
  - How many consensus sequences contain a deletion to the right of the cut site with or without insertions or microhomology?
- Insertion Count
  - How many consensus sequences contain an insertion with or without deletions?
- Microhomology Count
  - How many consensus sequences contain a microhomology signature with or without insertions?
- Normalized Microhomology
  - (Microhomology count) / (Scar Count)
- TMEJ
  - How many consensus sequences contain a Theta Mediated End Joining pattern?
- Normalized TMEJ
  - (TMEJ) / (Scar Count)
- NHEJ
  - How many consensus sequences contain a NHEJ signature?
- Normalized NHEJ
  - (NHEJ) / (Scar Count)
- Non-Microhomology Deletions
  - How many consensus sequences contain a deletion without a microhomology signature?
- Normalized Non-MH Del
  - (Non-Microhomology Deletions) / (Scar Count)
- Insertion >=5 +/- Deletions
  - Insertions ≥5 nucleotides with or without deletions.
- Normalized Insertion >=5+/- Deletions
  - (Insertion >=5 +/- Deletions) / (Scar Count)
- Other Scar Type
  - How many consensus sequences contain scars that do not match defined patterns?

ScarMapper Frequency File

This file contains information about the scar pattern seen in each consensus grouped by unique patterns. For this grouping, scar patterns are defined by the string “Left Deletions|Right Deletions|Microhomology|Insertion”.

1. Total
   1. Total number of times the pattern was observed in the sample.
2. Frequency
   1. Total number of times the pattern was observed normalized to the total number of reads in the library that passed filters.
3. Scar Type
   1. TMEJ: Theta signature End Joining
   2. HR: Homologous Recombination
   3. NHEJ: Non-Homologous End Joining
   4. non-MH Deletion: Deletion without microhomology
   5. Insertion: Insertions with or without deletions.
4. Left Deletions
   1. Nucleotides deleted to the left of the cut site.
5. Right Deletions
   1. Nucleotides deleted to the right of the cut site.
6. Deletion Size
   1. The size of the deletion is left + right + size of microhomology.
7. Microhomology
   1. Microhomology sequence.
8. Microhomology Size
   1. Size of microhomology.
9. Insertion
   1. Nucleotides inserted.
10. Insertion Size
    1. Size of insertion.
11. Left Template
    1. Nucleotides, based on insertions, that have homology within 50 nucleotides of the left junction.
12. Right Template
    1. Nucleotides, based on insertions, that have homology within 50 nucleotides of the right junction.
13. Consensus Left Junction
    1. Position of the left junction on the consensus sequence relative to the 5’ end.
14. Consensus Right Junction
    1. Position of the right junction on the consensus sequence relative to the 5’ end.
15. Target Left Junction
    1. Position of the left junction on the genomic target sequence relative to the 5’ end.
16. Target Right Junction
    1. Position of the right junction on the genomic target sequence relative to the 5’ end.
17. Consensus
    1. Consensus sequence.
18. Target
    1. Target region sequence.

References

1. Clement, K., et al., *CRISPResso2 provides accurate and rapid genome editing sequence analysis.* Nat Biotechnol, 2019. **37**(3): p. 224-226.

2. Taheri-Ghahfarokhi, A., et al., *Decoding non-random mutational signatures at Cas9 targeted sites.* Nucleic Acids Res, 2018. **46**(16): p. 8417-8434.

3. Brown, A.J., et al., *High-Throughput Analysis of DNA Break-Induced Chromosome Rearrangements by Amplicon Sequencing.* Methods Enzymol, 2018. **601**: p. 111-144.

**Part 2: PCR Conditions;**

# Lamin Receptor B PCR Conditions

1. 98.0 °C for 60 seconds
2. 98.0 °C for 15 seconds
3. 63.0 °C for 40 seconds; -0.5 °C per cycle
4. 72.0 °C for 45 seconds
5. Go To Step 2; 12 More Times
6. 98.0 °C for 15 seconds
7. 63.0 °C for 35 seconds
8. 72.0 °C for 50 seconds
9. Go To Step 6; 20 More Times
10. 72.0 °C for 300 seconds
11. 10 °C HOLD

# Illumina Dual Indexing PCR Conditions

1. 95 °C for 60 seconds
2. 95 °C for 30 seconds
3. 72 °C for 45 seconds
4. Go To Step 2; 6 more times
5. 72 °C for 5 minutes
6. 10 °C for HOLD

# PCR Conditions for Rosa26 Locus with Custom Primers

1. 98 °C for 30 seconds
2. 98 °C for 10 seconds
3. 62 °C for 30 seconds
4. 72 °C for 15 seconds
5. Go To Step 2; 2 more times
6. 98 °C for 10 seconds
7. 72 °C for 45 seconds
8. Go To Step 2; 23 more times
9. 72 °C for 2 minutes
10. 10 °C for HOLD

# PCR Conditions to Add Illumina Sequencing Adapters to Rosa26 Locus Custom Products

1. 98 °C for 30 seconds
2. 98 °C for 10 seconds
3. 65 °C for 30 seconds
4. 72 °C for 15 seconds
5. Go To Step 2; 6 more times
6. 10 °C for HOLD
7. 10 °C for HOLD

**Part 3: Step by step ddPCR.**

# Neon electroporation transfection

Day 1: Seed cells for Neon transfection;

Day 2: Neon transfection according to the user guide, in summary, aliquot 2.6 X 10^6^ cells in one 15 mL conical and wash the cells with PBS. Centrifuge the cells at 200g for 10 min followed by resuspend pellet in 130 µL Master Mix (5 µg Flag-Cas9, 5 µg sgRosa26, with or without 10 µg HR donor and 1 µg pEGFP-N2, resuspend in buffer R), using a 1350 V, 30 ms pulse in a 100 µl chamber (for MEFs).

Day 4: Collect cells for genomic DNA extraction 48 hours post transfection.

# Digital PCR

1. Prepare digital PCR reaction:

2 X ddPCR Supermix for Probes (No dUTP)

Forward primers (900 nM)

Reverse primers (900 nM)

Probe (250 nM)

Samples (80 ng)

Water to 25 µL.

Transfer 23 µL PCR reaction into a 96-well plate.

1. Generate droplet PCR by Automated Droplet Generator (Catelog #186-4101), after droplet generation, all the reactions will be in another 96-well plate. Seal the plates with the PX1 PCR Plate Sealer.
2. Proceed to thermal cycling according to the protocol.

Step 1: 95°C, 10 min, Ramp Rate 2°C/Sec;

Step 2: 94°C, 30 sec, Ramp Rate 2°C/Sec;

Step 3: 60°C, 30 sec, Ramp Rate 2°C/Sec;

Step 4: 72°C, 2 min, Ramp Rate 2°C/Sec;

Step 5: Go to Step 2, 39 cycles

Step 6: 98°C, 10 min, Ramp Rate 2°C/Sec;

Hold

1. After thermal cycling, place the sealed 96-well plate in the QX200 Droplet Reader. Open the QuantaSoft Software. Double click on a well in the plate layout to open the Well Editor dialog box. Write your sample name, experiment type, ddPCR Supermix for Probes (No dUTP) as the supermix type, target names and target types: Ch1 for FAM and Ch2 for HEX OR VIC. Select Apply and Ok when finish the setting.
2. After running the reader of the ddPCR, select samples in the well selector under Analyze. Use negative samples to set up a threshold to tell positive and negative signal.The concentration reported is copies/µl of the final 1 X ddPCR reaction.
